# Tuning the Circularly Polarized Reflection from Cholesteric Hydroxypropyl Cellulose Using Molecular Photoswitches

**DOI:** 10.1002/anie.202520839

**Published:** 2025-11-10

**Authors:** Fathy Hassan, Chun Lam Clement Chan, Thomas G. Parton, Zhen Wang, Qingchen Shen, Ruiting Li, Bruno Frka‐Petesic, Richard M. Parker, Silvia Vignolini

**Affiliations:** ^1^ Yusuf Hamied Department of Chemistry University of Cambridge Lensfield Road Cambridge CB2 1EW UK; ^2^ Department of Chemistry Faculty of Science Tanta University Tanta, El‐Gharbia 31527 Egypt; ^3^ Department of Sustainable and Bio‐inspired Materials Max Planck Institute of Colloids and Interfaces Am Mühlenberg 1 14476 Potsdam Germany; ^4^ International Institute for Sustainability with Knotted Chiral Meta Matter (WPI‐SKCM^2^) 1‐3‐1 Kagamiyama Hiroshima University Higashi‐Hiroshima Hiroshima 739–8526 Japan

**Keywords:** Cholesteric liquid crystals, Controlled polarization, Hydroxypropyl cellulose, Light‐responsive colors, Molecular switches

## Abstract

Controlling the ordering of molecular liquid crystals using light has found widespread applications in reflectors, sensors, and tunable optical filters. In chiral liquid crystals such as cholesterics, introducing a molecular switch can alter chiral interactions between mesogens and thus affect the periodicity and photonic band responsible for the structural color of the material. However, analogous photo‐control of cellulose‐based cholesteric polymers has not been reported. Here, we investigate the addition of achiral and chiral switches to cholesteric mesophases of structurally colored hydroxypropyl cellulose (HPC) and monitor changes in the photonic bandgap upon varying concentration, illumination, or temperature. While an achiral photoswitch enables reversible tuning of the reflected color upon *trans*‐*cis* photoisomerization, chiral switches (with different pendant groups and *R*/*S* enantiomers) lead to substantial differences in pitch and polarized reflection during cycles of illumination or heating/cooling–attributed to the complex interplay between the chiralities of the switch and of the host mesophase. A final combination of chiral photoswitch and spiropyran enables independent control of reflected and transmitted color, which can be relevant for cellulose‐based photonic displays, smart labels, or anti‐counterfeiting technology.

## Graphical abstract

Molecular photoswitches dispersed in a cholesteric hydroxypropyl cellulose mesophase enable reversible, light‐driven tuning of structural color and polarization. Enantiomer‐specific effects and light‐responsive patterning highlight the potential of dynamic, cellulose‐based photonic materials.

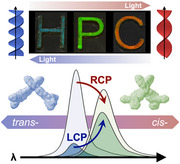

## Introduction

Hydroxypropyl cellulose (HPC) is a semi‐synthetic derivative of natural cellulose that is widely used in a range of commercial applications, from a bulking agent in food products to an excipient in the pharmaceutical industry.^[^ ^1^, ^2^, ^3^, ^4^, ^5^, ^6^ ^]^ As such, it already has many of the properties that are desirable to develop new sustainable, functional materials – in that it is biocompatible, biodegradable, low‐cost, and produced at a large scale.^[^ ^7^ ^]^ Similarly to other cellulose derivatives,^[^ ^8^ ^]^ HPC spontaneously arranges into a polymeric liquid crystalline phase in solution.^[^ ^9^, ^10^, ^11^ ^]^ Specifically, when HPC is dissolved at ambient temperature and at high concentrations into a polar solvent, such as water or dimethyl sulfoxide (DMSO), it self‐organizes into a lyotropic cholesteric mesophase.^[^ ^12^, ^13^, ^14^, ^15^, ^16^ ^]^ The combination of this long‐ranged helicoidal nanostructure with the intrinsic birefringence of HPC leads to a selective reflection of circularly polarized light, centered at a wavelength determined by the pitch *p* of the periodic cholesteric structure.^[^ ^17^, ^18^, ^19^ ^]^ This vibrant “structural coloration” makes HPC an ideal candidate for a wide range of applications, from incorporation as an edible photonic colorant,^[^ ^20^, ^21^, ^22^ ^]^ to use as a colorimetric sensor for external stimuli, including strain,^[^ ^23^, ^24^ ^]^ pressure^[^ ^25^, ^26^ ^]^ or temperature.^[^ ^27^ ^]^ However, to achieve the additional functionalities required for more dynamic photonic applications, it is desirable to be able to tune the reflected coloration without a mechanical stimulus. In this context, using light to switch the optical response offers multiple advantages, such as remote control and low energy consumption.^[^ ^28^, ^29^, ^30^ ^]^

Doping molecular liquid crystals with photoresponsive molecules is a well‐established strategy to remotely tune their optical and mesophase properties, with a variety of systems studied to date.^[^ ^31^, ^32^, ^33^, ^34^, ^35^, ^36^ ^]^ However, this formulation‐based approach has not yet been investigated for cellulosic polymers due to challenges in compatibility between the dopant and polymer matrix. Instead, previous studies have focused on covalently functionalizing HPC with azobenzene moieties to produce photoresponsive polymers for applications including: a tunable phase transition,^[^ ^37^ ^]^ photochromic and thermotropic liquid crystalline behavior,^[^ ^38^ ^]^ inducing a photoregulated sol‐gel transition,^[^ ^39^ ^]^ or for controlling their surface properties,^[^ ^40^ ^]^ or their light‐induced solubility.^[^ ^41^ ^]^

**Figure 1:**
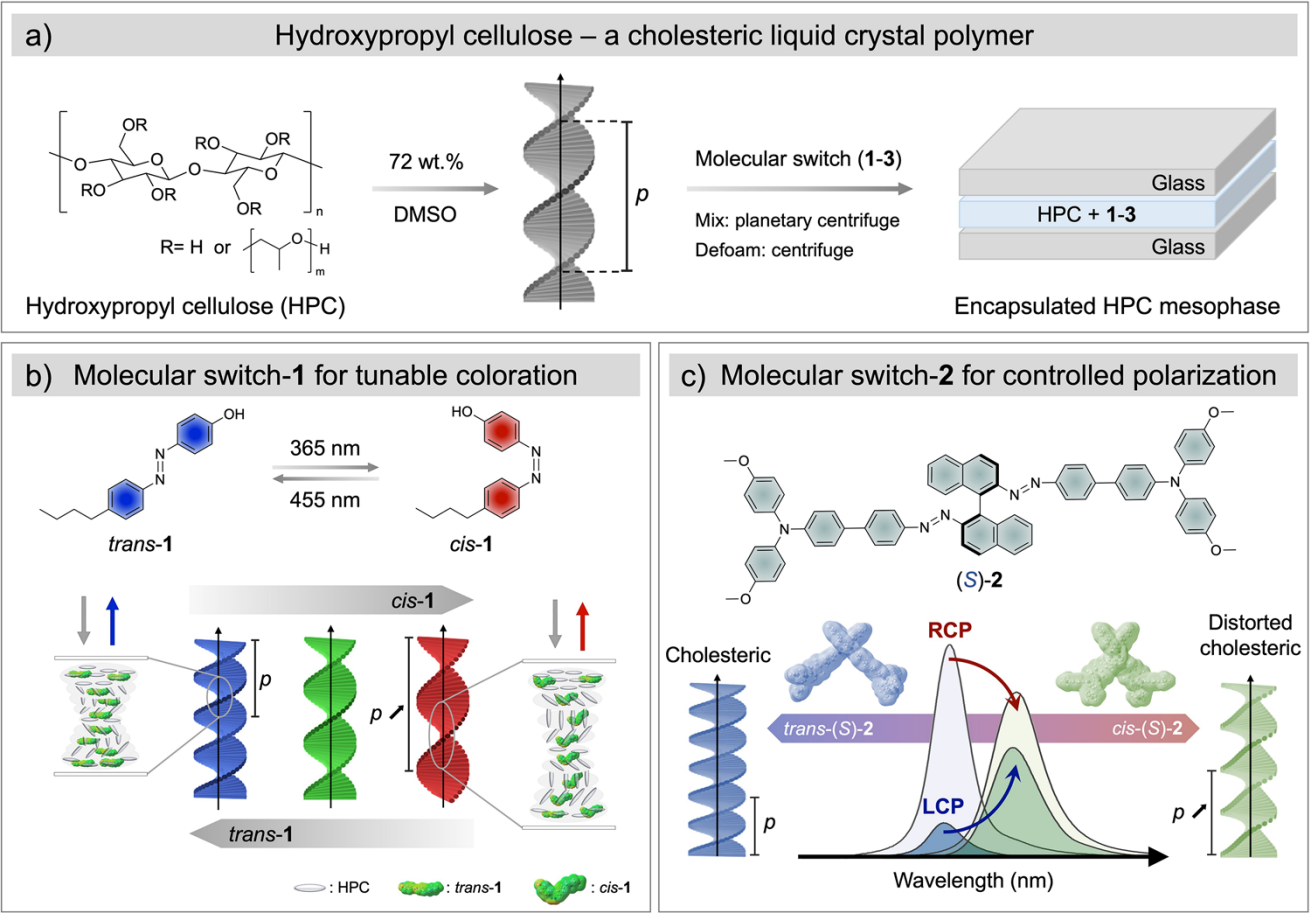
Photoresponsive molecular switches are used to tune the optical response of an encapsulated hydroxypropyl cellulose (HPC) mesophase. a) The semi‐synthetic polymer HPC can self‐organize into a structurally colored liquid crystalline phase in DMSO, where the reflected wavelength is determined by the pitch, *p* of this cholesteric phase. The HPC mesophase can be formulated with different molecular switches (**1**–**3**) to achieve dynamic optical responses. b), Achiral molecular switch **1** can be photoisomerized from the *trans* to the *cis* isomer, with a corresponding increase in the cholesteric pitch of the HPC mesophase. This allows for the reflected color to be smoothly and reversibly tuned across the visible spectrum from blue to red wavelengths. c) Chiral molecular switch **2** additionally allows for the polarization of the reflected color to be tuned by inducing distortion into the cholesteric helix, as exemplified by the reflection spectra for the visible light‐controlled polarization by (*S*)**‐2** (0.1 wt.%). The optimized molecule geometry with electron density distribution map was obtained from density functional theory (DFT) at B3LYP 6‐31G level.

In this study, we investigate the potential of directly doping an HPC mesophase with various photoresponsive molecular switches to tune its cholesteric structure and, thus, its optical properties (Figure 1a). To explore the effect of conformational change upon isomerization, three different molecular switches were selected, as summarized in Scheme S1). These were: i) an achiral amphiphilic azobenzene **1**, ii) enantiomers of a chiral binaphthyl‐azo, with two different extended structures, yielding (*R*)‐**2**, (*S*)‐**2**, and (*R*)‐**2**′, (*S*)‐**2**′, and iii) photochromic spiropyran (SP‐**3**), which can reversibly photoisomerize to merocyanine (MR‐**3**). These molecular switches **1**–**3** were mixed into solutions of HPC (HPC SSL SFP, *M*_W_ = 40000 g mol^−1^, Nisso Chemical Europe) at 72 wt.% in DMSO and encapsulated between glass microscope slides with a gap of 1 mm for optical measurements (see Methods and Figures S1–S3). Within this cell, the HPC solution spontaneously organizes into a polydomain cholesteric structure.^[^ ^22^, ^24^, ^42^, ^43^, ^44^, ^45^ ^]^ The behavior of these mesophases was then investigated under photoisomerization cycles, as well as under control conditions, such as exposure to moderate heating or cooling, to account for their thermotropic behaviors.

We found that incorporation of 0.1 wt.% of **1** enables reversible tuning of the HPC reflectance peak across the visible spectrum, owing to an increase in cholesteric pitch upon *trans‐cis* photoisomerization (Figure 1b). Moreover, when 0.1 wt.% of **2** or **2**′ was instead added to the formulation, the polarization state of the reflected light could be modified, depending on the specific enantiomer added (Figure 1c). Finally, we show that an encapsulated HPC mesophase containing a combination of photoswitch (*S*)‐**2**′ and photochromic spiropyran **3** can be spatially patterned by exploiting their different illumination wavelengths and coloration mechanisms. The unique optical signature that such an approach can produce exemplifies the potential of the HPC/photoswitch system for applications including low‐energy displays and security labeling technology.

## Results and Discussion

### Tuning the Color of Cholesteric HPC Using an Achiral Molecular Switch

To explore if the pitch and thus the reflected color of an HPC mesophase could be tuned via a light‐responsive molecular switch, we first considered 4‐(4‐butylphenylazo)phenol (**1**, Figure 1b), a simple amphiphilic achiral azobenzene compound that is soluble in polar solvents. We used UV–vis spectroscopy to confirm that **1** undergoes efficient and reversible *trans‐cis* photoisomerization (Figure S4a). Illumination with ultraviolet (UV) light (*λ*_I_ = 365 nm, *I* = 40 mW cm^−2^) led to a significant decrease in the intensity of the absorption peak at 320 nm (corresponding to the π–π* transition of the *trans* isomer), and an increase in the absorbance at 440 nm (corresponding to the n‐π* transition of the *cis* isomer). A photostationary state (PSS) in solution was reached after 5 min of 365 nm illumination with a *trans*:*cis* ratio of 35:65. Illumination with blue light (*λ*_I_ = 455 nm, *I* = 20 mW cm^−2^) rapidly drove the reverse *cis*‐to‐*trans* photoisomerization, reaching a PSS close to the initial state (*trans*:*cis* ratio 94:6) within 5 min. This switching behavior was consistent over ten cycles of alternating illumination (Figure S4b), confirming good fatigue resistance.

**Figure 2:**
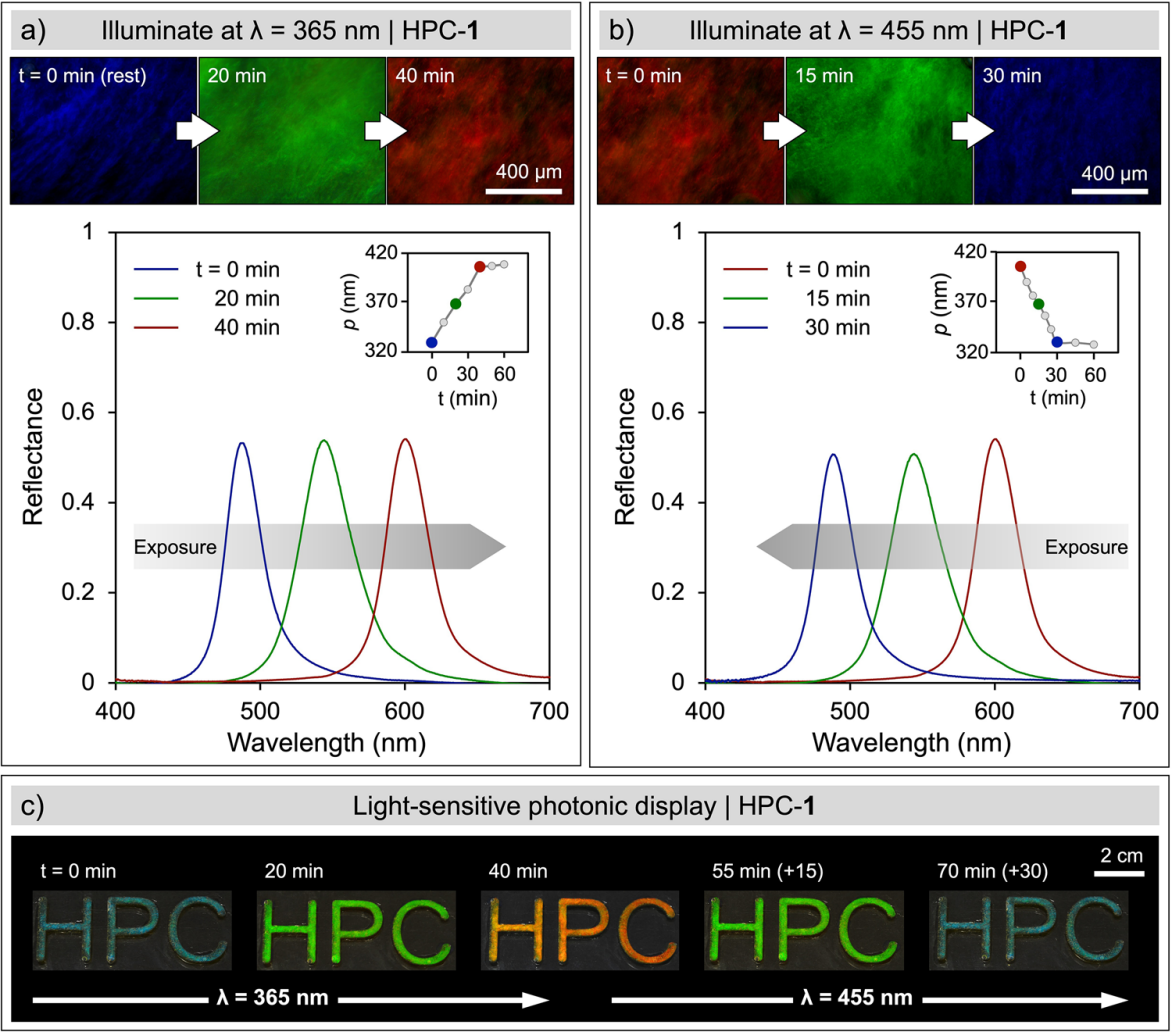
Optical microscope images (dark‐field reflection) and corresponding micro‐spectra of achiral photoswitch **1** at 0.1 wt.% in an HPC mesophase upon a), UV light illumination (365 nm, 40 mW cm^−2^) followed by b), visible light illumination (455 nm, 20 mW cm^−2^). The inset plots show the relation between the pitch (*p*) and the illumination time, allowing for the PSS to be identified. c), Light‐sensitive photonic display prepared from HPC‐**1** encapsulated within a patterned substrate.

We then doped an HPC solution (72 wt.%, DMSO) with molecular switch **1** at a range of concentrations. Optical microscopy and micro‐spectroscopy (Methods, Supporting Information) demonstrated that by increasing the concentration of **1** within the mesophase from 0.1 to 2.0 wt.%, the reflected color could be tuned from blue (*λ*_max_ = 495 nm) to red (*λ*_max_ = 605 nm), which is attributed to an increase in the cholesteric pitch (Figure S5). More strikingly, we then found that a comparable magnitude of color shift could be achieved in an HPC mesophase containing only 0.1 wt.% of **1** by simply illuminating it with UV light (*λ*_I_ = 365 nm, *I* = 40 mW cm^−2^). As shown in Figure 2a, continuous UV exposure results in a linear redshift of the reflected color until the photostationary state (PSS_365nm_) is reached after approximately 40 min. This color shift could then be reversed by illuminating the sample with blue light (*λ*_I_ = 455 nm, *I* = 20 mW cm^−2^), with the initial reflected blue color restored after 30 min (Figure 2b). The color switching of the HPC mesophase shows good reproducibility over five cycles of alternating illumination at *λ*_I_ = 365 and 455 nm (Figure S6), suggesting that this effect is driven by the photo‐isomerization of **1**. The peak reflection wavelength *λ*_max_ of a cholesteric mesophase is related to the pitch *p* by the equation *λ*_max_ = *n* *p*, where *n* is the average refractive index. For 72 wt.% HPC in DMSO, the refractive index was estimated to be *n* ≈ 1.49 (Equation S1), assuming the low concentration of **1** has a negligible contribution.^[^ ^46^ ^]^ As such, the color shifts upon UV and blue illumination can be directly attributed to the change in cholesteric pitch (insets of Figure 2a,b) arising from the conformational change of the azobenzene moiety of **1** upon photoisomerization.

To understand this dynamic pitch change, we first considered the possible role of temperature, as HPC mesophases are known to display thermochromic behavior (reported as approx. +12 nm/°C in water).^[^ ^27^ ^]^ We therefore introduced a thermocouple into the glass characterization cell (Methods, Supporting Information) to monitor the in situ temperature change in the HPC mesophase. For a pure HPC mesophase, exposure with UV light (*λ* = 365 nm) for 1 h resulted in slight heating of the sample (Δ*T*_I_ ≤ 0.8 °C) and a reflection peak shift of Δλ_max_ = +10 nm (Figure S7). Direct heating of an equivalent HPC sample by 0.8 °C on a temperature‐controlled stage led to a similar peak shift (Figure S7). This indicates that the peak shift under UV illumination for pure HPC can be solely attributed to thermotropic behavior (i.e., Δλ_max_/Δ*T*_H_ ≈ +12.5 nm/°C in DMSO, Figure S24).

For the HPC‐**1** mixture, a much greater redshift was observed upon UV illumination (Δλ_max_ = +110 nm) while the temperature increase remained small (Δ*T*_I_ ≈ 1.2 °C over the first 15 min, before reaching a plateau for the remainder of the 50 min illumination period, Figure S8). However, direct heating of the HPC‐**1** mixture by a similar amount (Δ*T*_H_ ≈ 1.7 °C) induced only a shift of Δλ_max_ = +22 nm, suggesting the thermochromic behavior remains comparable to pure HPC (Figures S9 and S24). As such, this indicates that the UV‐induced red‐shift dominates the heat‐induced color change (Δλ_max_/Δ*T* ≈ +100 ± 17 nm/°C), with a thermotropic contribution to the observed red‐shift being no more than 13 ± 2.5% (Table S1).

To understand this effect, we considered the role of molecular switch conformation on the HPC mesophase. DFT models of the *trans* and *cis* isomers of **1** (Table S2) show that the geometry of the initial *trans* isomer is planar and has a convex hull volume *V*_ch_ = 343.3 Å^3^ (the convex hull being the smallest convex envelope containing the molecule). However, the *cis* isomer has a bent conformation with an axial twist between the phenyl rings and a convex hull volume *V*_ch_ = 412.3 Å^3^. As such, the less compact conformation of the *cis* isomer may be responsible for disrupting the hydrogen‐bonding network between HPC chains, thus weakening the effective chiral interaction strength.^[^ ^47^, ^48^ ^]^

To further understand how conformational changes can influence the pitch, we also prepared HPC mesophases containing SP‐**3**, which can undergo closed‐open ring photoisomerization to form MR‐**3**. UV–vis spectroscopy confirmed that 3 min of UV illumination (*λ*_I_ = 365 nm, *I* = 40 mW cm^−2^) was sufficient to drive the transition from an initial SP‐**3** state, which only strongly absorbs below 400 nm, to a MR‐**3** state with a new maximum absorption band at 560 nm that corresponds to an intense magenta color (Figure S10). SP‐**3** (0.1 wt.%) was then introduced into an HPC mesophase following the same protocol as for **1**. Upon UV illumination (*λ*_I_ = 365 nm, *I* = 40 mW cm^−2^), the reflected color shifted from blue (*λ*_max_ = 498 nm) to green (*λ*_max_ = 532 nm) after 20 min of UV illumination, after which no further shift was observed (Figure S11a). The magnitude of this redshift (Δλ_max_ = +34 nm) and the associated temperature increase (Δ*T* ≈ 2.1 °C) remains moderate and ascribable to radiative heating (Δλ_max_/Δ*T* ≈ +16 nm/°C), as further confirmed by the redshift induced simply by heating the HPC‐**3** mesophase (Δλ_max_ = +34 nm for Δ*T* ≈ 2.1 °C, also yielding Δλ_max_/Δ*T* ≈ +16 nm/°C, Figure S11d). As such, this is consistent with a solely thermotropic effect, meaning that the conversion from SP‐**3** to MR‐**3** itself is not responsible for the increase in pitch. In contrast to the *trans*‐to‐*cis* isomerization of compound **1**, the ring‐opening reaction of SP‐**3** to form MR‐**3** results in only a slight change in convex hull volume *V*_ch_ (from 440.9 Å^3^ for SP‐**3** to 435.9 Å^3^ for MR‐**3**; Table S3 and Section S14).

As a demonstration of this tunable system, we laser‐cut the letters “H P C” into a PMMA substrate, filled it with the HPC‐**1** solution, and sealed it with a layer of glass (Figure 2c). By reversibly illuminating the sample with UV and then blue light, the reversible “RGB” color tuning is clearly visible to the naked eye under ambient conditions. A benefit of using HPC as the host liquid crystalline phase is that its organization into a cholesteric state is spontaneous rather than induced by the addition of a chiral dopant. The resulting color tunability is then readily achieved through the incorporation of a single, nonchiral dopant such as **1**. In that aspect, it is distinct from previous studies,^[^ ^49^, ^50^, ^51^, ^52^, ^53^ ^]^ where enantiomerically pure chiral dopants were necessary to both induce cholesteric order from a nematic molecular liquid crystal (such as E7) and also to tune the resultant pitch to reflect at specific visible wavelength bands.

### Controlling the Degree of Circular Polarization Using a Chiral Molecular Switch

To explore the role of stereoisomerism on the interaction between the photoswitch with the cholesteric phase, we synthesized two similar molecular switches based on a chiral azobinaphthyl core (**2** and **2**′), each in two enantiomerically pure samples. While similar molecular switches have been used as a chiral dopant for small molecule nematogens,^[^ ^49^, ^50^, ^51^, ^52^, ^53^ ^]^ HPC has an intrinsic tendency to form a chiral mesophase. As such, the interactions between the chiral dopant and the chiral host can either enhance or diminish the initial twist, leading to divergent properties. Molecular switches **2** and **2**′ were selected because they contain both a photoresponsive azobenzene moiety and a binaphthyl moiety as an axially chiral segment in a single structure, resulting in a photoinduced chiral switching property. Moreover, by starting with either the (*R*)‐(+) or (*S*)‐(−) enantiomer of 1,1′‐binaphthyl‐2,2′‐diamine (Scheme S2), we could synthesize (*R*)‐**2** and (*R*)‐**2**′, and (*S*)‐**2** and (*S*)‐**2**′, which have the opposite handedness. An additional benefit of using this class of molecular switches is the ability to tune the illumination wavelengths used for photoisomerization. The direct attachment of aromatic groups at the *para* positions of the chiral azobinaphthyl core extends the π‐conjugation in the molecule and, therefore, enables both forward and backward switching using visible light.^[^ ^54^ ^]^ Specifically, molecular switch **2** uses a triphenyl amine segment, which enables visible light‐driven switching at 455 nm for *trans‐cis* isomerization and 520 nm for *cis‐trans* isomerization (Figure S12). Alternatively, molecular switch **2**′ uses a pentylbiphenyl segment, which can be switched at 405 nm and 455 nm, respectively (Figure S13). We confirmed that, unlike for UV illumination, switching **2** and **2**′ using visible light leads to negligible heating of the HPC mesophase (Δ*T* ≤ 0.2 °C after 30 min, Figure S14).

Circular dichroism (CD) measurements of **2** and **2**′ in 1,4‐dioxane were used to evaluate their chiral induction ability (Figure S15). For both molecular switches, *trans*‐*cis* isomerization led to a decrease in the CD peaks around 220 and 320 nm, which can be attributed to the ^1^B_b_ and ^1^L_b_ transitions of the 1,1′‐binaphthyl with a photomodulation of the dihedral angle (19% and 24% from the initial state for compounds **2** and **2**′, respectively).^[^ ^55^ ^]^ Exciton couplets attributed to the π–π* transition of the aryl azo group are also observed at approximately 460 and 410 nm for **2** and **2**′, respectively, while exciton couplets attributed to the n‐π* transition of the aryl azo group are observed at 520 and 470 nm, respectively. The exciton couplets of π‐π* are significantly decreased, and the n‐π* increased upon *trans‐cis* photoisomerization (*see the DFT models*, Tables S4 and S5). This switching behavior was reversible and reproducible over multiple illumination cycles (Figure S16)

HPC mesophases were then prepared by doping with each of the four chiral molecular switches (*R*)‐**2**, (*R*)‐**2**′, (*S*)‐**2**, and (*S*)‐**2**′ following the same protocol as for **1**. First, comparing each pair of (*R*) and (*S*) enantiomers, the peak reflection from molecular switch **2**′ is redshifted by approx. 20 nm relative to **2** (Figure S17). Second, it is interesting to note that before illumination there is a distinct color difference between mesophases containing the (*R*) and (*S*) enantiomers at the same concentration: at 0.1 wt.% the (*S*) enantiomers reflected blue color, while the corresponding (*R*) enantiomers had larger equilibrium pitches resulting in green reflected color. The difference in peak reflectance wavelength between (*R*) and (*S*) enantiomers diverges as the concentration of the molecular switch increases, with the (*R*) enantiomers displaying a much stronger red‐shifting effect on the HPC mesophase. Such differences can arise from the enantiospecific interactions between the molecular switches and HPC. Furthermore, these interactions can be further amplified by any differences in conformation between the (*R*) and (*S*) isomers within a chiral medium. In particular, the conformation of the binaphthyl rings, characterized by its dihedral angle (*θ*), has been demonstrated to greatly impact chirality amplification in supramolecular systems.^[^ ^56^, ^57^, ^58^, ^59^, ^60^ ^]^ In small molecule cholesteric liquid crystals, doping with (*S*) enantiomers typically induces a right‐handed helix when 0° < *θ* < 90°, whereas they induce left‐handed helicity when 90° < *θ* < 180° (Section S14).^[^ ^50^, ^61^ ^]^ Note that the dependence of the pitch on the concentration of additives could, in principle, allow for the extraction of scaling factors, following *p* = 2π *K*_22_(*c*) /*K_t_* (*c*) where *K*_22_ and*K_t_* are the twist elastic constant and the chiral strength of the mesophase, respectively (Figure S18). However, the upper spectral limit of our micro‐spectroscopy setup (*ca*. 800 nm) and the limited number of available data points at high loadings (constrained by the upper solubility limit of the additives) prevented us from reliably estimating these scaling constants (see Table S6 and Section S10). An attempt to improve the estimation of these parameters was explored by merging the two datasets for **2** and **2**′ assuming comparable relative photoswitching effects, *K_t_*, and the chiral strength of the mesophase, *K*_22_. This analysis suggests that the chirality of the HPC and the dopants interferes strongly, as *K_t_* displays both a symmetric contribution (i.e., common to (*R*) and (*S*)) and an antisymmetric contribution (i.e., opposite for (*R*) and (*S*)) from the dopant (Section S10).

Looking beyond simply tuning the cholesteric pitch of the HPC mesophase, we also considered whether the chirality of the molecular switch may affect the polarization of the optical response. A well‐ordered cholesteric HPC domain has a right‐handed cholesteric structure and should, therefore, reflect right‐circularly polarized (RCP) light at normal incidence.^[^ ^62^, ^63^ ^]^ However, disorder within the cholesteric structure, or illumination at oblique angles of incidence with respect to the cholesteric helical axis, can lead to partial reflection of left‐circularly polarized (LCP) light.^[^ ^64^, ^65^ ^]^ To measure the polarized optical response from our samples, we introduced a linear polarizer and a quarter‐wave plate into our microscopy and micro‐spectroscopy setup to collect LCP or RCP reflection images and spectra (Methods, Supporting Information). Using the LCP and RCP reflectance at the peak wavelength, λ_max_, we also evaluated the degree of circular polarization (DCP), given by:

(1) $$\text{DCP}=\frac{I_{\mathrm{RCP}}-I_{\mathrm{LCP}}}{I_{\mathrm{RCP}}+I_{\mathrm{LCP}}}$$

**Figure 3:**
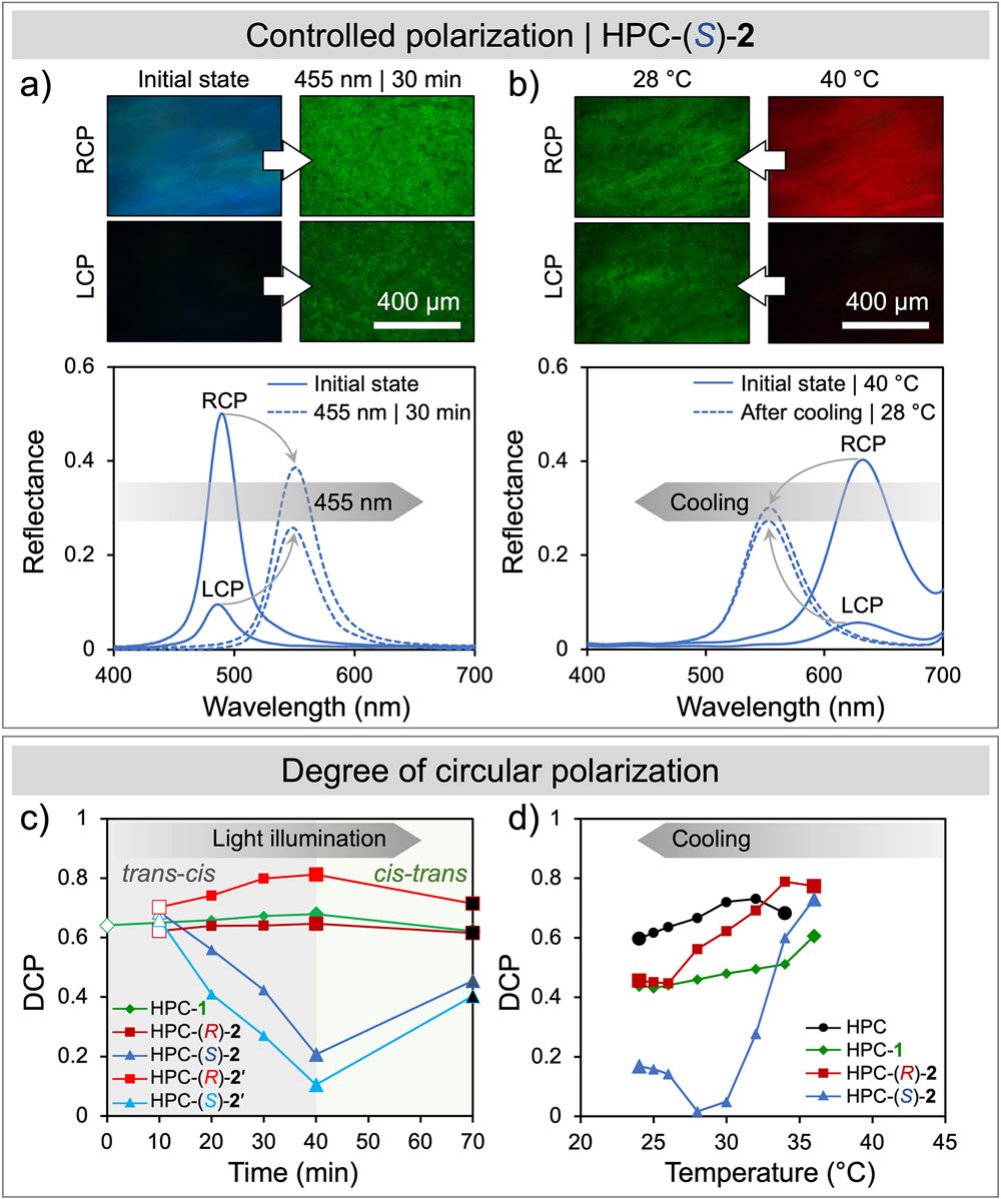
a), Polarized optical microscopy (POM) images (dark‐field reflection) and corresponding micro‐spectra of chiral photoswitch (*S*)‐**2** at 0.1 wt.% in the HPC mesophase upon visible light illumination. Solid lines for the initial states and dashed lines for the PSSs after illumination at 455 nm. b), POM images (dark‐field reflection) and corresponding micro‐spectra of (*S*)‐**2** at 0.1 wt.% in the HPC mesophase upon cooling. Solid lines for the initial states and dashed lines upon cooling. c), Degree of circular polarization (DCP) for molecular switch **1**, (*R*) and (*S*) enantiomers of **2** and **2**′ after light‐induced *trans*‐to‐*cis* isomerization (Hollow symbols represent the initial states, enlarged filled symbols indicate the PSSs, and solid black symbols mark the final points after the *trans*–*cis* back reaction). d), The change in DCP upon cooling for pure HPC (control experiment), and HPC containing either molecular switch **1** or (*R*) and (*S*) enantiomers of **2**.

The time evolution of DCP upon isomerization is summarized in Figure 3c. As an initial test, we measured the polarized reflectance from an HPC mesophase doped with the achiral molecular switch **1** during UV illumination (Figure S19). The reflection was predominantly RCP throughout the *trans*‐*cis* color shift (DCP = + 0.65, close to the ideal value of + 1 for a right‐handed cholesteric at normal incidence), suggesting that photoisomerization of **1** changes the pitch without disrupting the cholesteric ordering. We then measured the polarized reflectance from HPC mesophases doped with the chiral molecular switches upon *trans‐cis* isomerization. The HPC‐(*R*)‐**2** mesophase initially exhibited green RCP‐dominated reflection (λ_max_ = 540 nm, DCP = + 0.60) that red‐shifted upon isomerization with no change in DCP (λ_max_ = 610 nm, DCP = + 0.60), similar to the HPC‐**1** mixture (Figure S20). However, the HPC‐(*S*)‐**2** mesophase showed remarkably different behavior (Figure 3a): although the mixture initially exhibited strong RCP‐dominated blue reflection (λ_max_ = 490 nm, *I*_RCP_ = 0.50, DCP = + 0.69), the red‐shift upon *trans‐cis* isomerization (*λ*_I_ = 455 nm, 20 mW cm^−2^) was accompanied by a decrease in peak RCP reflectance and an associated increase in LCP reflectance (λ_max_ = 550 nm, *I*_RCP_ = 0.39, DCP = + 0.21), as well as the emergence of a finer‐grained texture in the optical microscopy images that was not seen for the HPC‐(*R*)‐**2** mesophase (Figure S20). Similar behavior was observed when comparing HPC mesophases doped with the (*R*) and (*S*) isomers of molecular switch **2**′ when *trans‐cis* isomerization was driven by violet light (*λ*_I_ = 405 nm, *I* = 25 mW cm^−2^, Figure S21). For both chiral switches, there was a significant decrease in DCP for HPC mesophases doped with the (*S*) enantiomers, while DCP slightly increased for those doped with (*R*) enantiomers. As such, we propose that photoisomerization of (*S*)‐**2** or (*S*)‐**2**′ leads to increasing distortion of the cholesteric helix,^[^ ^66^ ^]^ as previously reported for other cholesteric systems.^[^ ^67^, ^68^ ^]^

To investigate whether the decrease in DCP upon *trans‐cis* isomerization was related to the use of visible illumination wavelengths, we exploited the fact that UV illumination of **2**′ (*λ*_I_ = 365 nm) could also efficiently induce *trans*‐*cis* isomerization, with a PSS spectrum almost indistinguishable from that obtained after violet (λ_I_ = 405 nm) illumination (Figure S22). However, UV illumination of an HPC‐(*S*)‐**2**′ mesophase instead led to a slight increase in DCP, as well as a greater red‐shift than that observed for 405 nm illumination, from λ_max_ = 485 nm and DCP = + 0.66 to λ_max_ = 610 nm and DCP = + 0.72 after 40 min of UV illumination (Figures S23a and S27c,e). Intriguingly, subsequent illumination of the HPC‐(*S*)‐**2**′ mesophase with visible light (*λ*_I_ = 455 nm) to drive *cis‐trans* isomerization then led to a strong decrease in DCP and the emergence of a finer‐grained microscopic texture, that will be discussed further below (Figures S23b and S27c,e).

To explore the role of temperature in these DCP changes, we performed polarized micro‐spectroscopy of HPC mesophases while heating the samples using a hotplate (Δ*T*_H_ ≈ 14 °C) and then allowing them to cool back to ambient temperature (Figure S24). The heat‐induced redshift was found to be between 9 and 13 nm/°C across different HPC formulations and heating/cooling cycles. In practice, this means that the internal temperature increase required to tune the color from blue to red is about 13–18 °C for HPC mesophases (Figures S24 and S25 for the particular case of HPC‐(*S*)‐**2**). As shown in Figure S27a, there was no significant change in DCP upon heating (DCP = + 0.75). However, there were clear differences between samples upon cooling (Figures 3d and S27d): the pure HPC mesophase returned to its original DCP, whereas HPC‐**1**, HPC‐(*R*)‐**2**, and HPC‐(*S*)‐**2** exhibit significant DCP hysteresis, with substantial drops in DCP during cooling. The most dramatic difference is displayed by the HPC‐(*S*)‐**2** sample (Figure 3b), where the DCP dropped to a transient value of DCP ≈ 0, indicating near‐equal RCP and LCP intensities, before reaching a final value of DCP = +0.2 as the sample returned to its original temperature (Figures 3d and S26b). To facilitate comparison between illumination and heating‐cooling cycles, the DCP variation with respective to the relative redshift is plotted in Figure S27e,f, which clearly illustrates that the hysteresis of DCP upon heating‐cooling is much stronger than upon photoswitching, with a marked DCP drop for HPC‐(*S*)‐**2** compared to HPC‐(*R*)‐**2** in both cases (Figure S27b–d).

For polydomain cholesteric samples, a decrease in DCP can arise from local distortion of the helicoidal structure (i.e., non‐sinusoidal variation of the local director with respect to the helical axis), or from misalignment of the helical axis with respect to the sample axis, both of which cause local interconversion of LCP and RCP light.^[^ ^69^, ^70^, ^71^ ^]^ In HPC mesophases, a drop in DCP and decrease in pitch upon partial water evaporation was previously ascribed to spontaneous Helfrich‐Hurault (HH) elastic instabilities, which occur when the local pitch contraction results in the buckling of large‐scale cholesteric texture into sheared and tilted domains, resulting in a pattern at length scales much larger than the pitch.^[^ ^20^, ^72^ ^]^ In cholesteric phases of thermotropic polypeptides, patterns consistent with HH elastic instability have been also observed exclusively upon pitch contraction (induced by a sudden temperature drop).^[^ ^73^ ^]^ In our system, a transient DCP decrease was observed upon both heating and cooling, but the DCP drop upon pitch relaxation toward smaller values (which could be explained by the HH instability) appeared nearly an order of magnitude faster than the DCP drop upon pitch relaxation toward larger values, and was also longer‐lasting. While an elastic instability can explain the DCP drop upon pitch decrease (i.e., during cooling) and the finer‐grained microscopic texture mentioned above for HPC‐(*S*)‐**2** and HPC‐(*S*)‐**2′** (Figure S27e), it does not explain why the DCP drop is consistently more pronounced for the (*S*)‐**2** and (*S*)‐**2′** enantiomers compared to their (*R*) counterparts. These observations lead us to conclude that an alternative mechanism is responsible for the origin of the DCP drop upon photoswitching, and that its main origin is a distortion of the helicoidal modulation due to different chiral interactions at the molecular level between the HPC mesophase and the photoswitches. To further explore this interpretation, we provide a dedicated discussion of this mechanism involving the dependencies of the chiral strength term χ and the twist elastic term κ and relate them to the molecular morphological parameters of the different dopants (Section S14).^[^ ^74^, ^75^ ^]^

### Light‐Responsive Patterning

**Figure 4:**
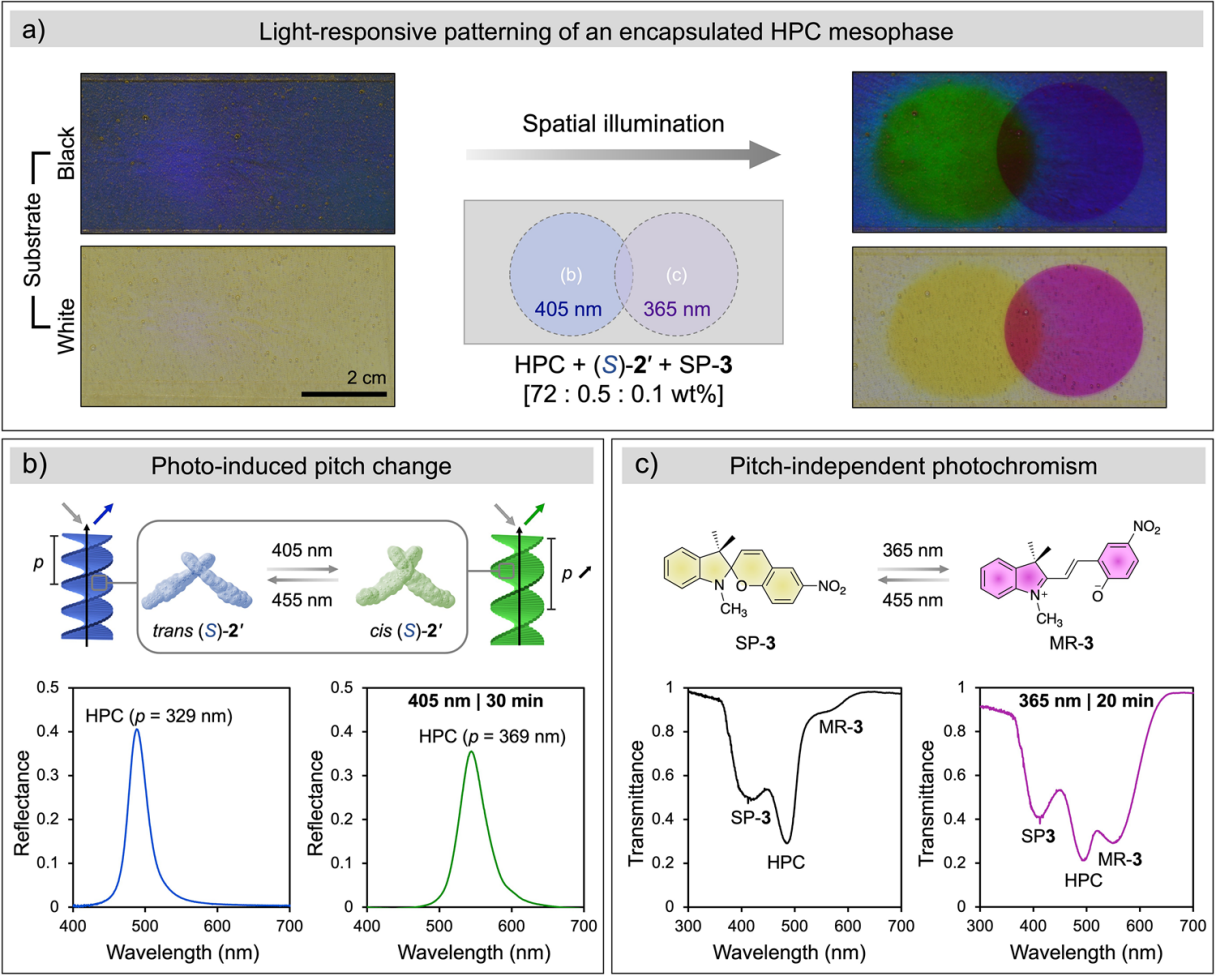
Light‐responsive patterning of an encapsulated HPC mesophase. a), Photographs of a cell containing a mixture of (*S*)‐**2**′ (0.25 wt.%), **3** (0.05 wt.%)and HPC (72 wt.%) in DMSO when viewed on both a white and black substrate and taken before and after illumination with 405 and 365 nm light in two overlapping disks. b), Photo‐induced pitch change owing to *trans*‐*cis* isomerization of (*S*)‐**2**′ and the corresponding change in reflection spectra upon 405 nm light illumination. The optimized geometry with electron density distribution map was obtained from DFT at B3LYP 6‐31G level. c), Pitch‐independent photochromism of SP‐**3** and the corresponding change in the transmission spectra owing to the photoinduced ring‐opening to MR‐**3**.

Photochromic molecules such as spiropyran^[^ ^76^, ^77^ ^]^ have recently shown promise as a way to produce rewritable images for authentication and security purposes^[^ ^78^, ^79^, ^80^ ^]^ or for “ink‐free paper”.^[^ ^81^ ^]^ In all these applications, it is necessary to inhibit diffusion of the dye to ensure a well resolved image. We therefore investigated the use of high‐viscosity HPC mesophases as the matrix for SP‐**3**. We observed that localized exposure to UV light (via either a photomask or by direct writing using a focused light beam) can be used to spatially resolve patterns in the HPC mesophase (respectively Figures S28 and S29). Moreover, by combining this effect with the structural coloration of the cholesteric mesophase, the system allows for a second layer of addressability. As a practical demonstration of the untapped potential for phototunable HPC mesophases in such applications, we prepared a larger cell containing a mixture of both molecular switch (*S*)‐**2**′ and photochromic SP‐**3** in a 5:1 ratio. Natively, this mesophase reflects light around *λ*_max_ = 490 nm, while all other wavelengths are transmitted. Under ambient illumination, this results in the cell appearing blue when placed on an absorbing, black substrate and almost colorless when placed on a scattering, white substrate (Figure 4a). Next, we illuminated two overlapping circular regions of the cell with violet or UV light. Within the left circle illuminated with violet light (*λ*_I_ = 405 nm, *I* = 25 mW cm^−2^), photoisomerization of (*S*)‐**2**′ resulted in an increase in the pitch of the HPC mesophase and a corresponding localized shift in the reflected color to green (*λ*_max_ = 550 nm), with a complementary yellow appearance when viewed on a white substrate (Figure 4b). In contrast, the right circle illuminated with UV light (*λ*_I_ = 365 nm) showed a strong magenta coloration with a white substrate (*cf*. transmission), but little change on a black substrate (*cf*. reflection). This can be explained by the colorless spiropyran undergoing closed‐open ring isomerization to merocyanine (which absorbs strongly at 550 nm, leading to magenta coloration in transmission), without significant impact on the pitch of the HPC mesophase and thus the reflected color (Figure 4c). In the overlapping region, both molecular switches independently underwent photoisomerization, resulting in a combination of both visual effects. Finally, by exposing the entire cell to visible blue light (*λ*_I_ = 455 nm), the initial state could be reset, ready for a different pattern to be applied.

## Conclusions

In this study, we explored how the photonic properties of a chiral HPC mesophase can be modulated in terms of color shift and polarization change, upon illumination or heating‐cooling cycles in the presence of achiral or chiral photoswitches. The use of a simple achiral azobenzene derivative demonstrates how the photonic bandgap can be tuned reversibly and repeatably across the visible spectrum using only light. A richer variety of mesophase behavior and optical response was observed when combined with chiral switches. The strikingly different polarization change upon illumination using either visible or UV light was ascribed to an underlying effect of UV‐induced heating, combined with the thermochromism of the HPC matrix, which caused hysteresis of its DCP via a Helfrich‐Hurault instability. The use of visible wavelengths with negligible heating led to a reversible and reproducible tuning of the bandgap and its DCP (ascribed to a distortion of the cholesteric helical modulation) that appears to be enantiomspecific. This allowed for the demonstration of a facile method to produce tunable structural coloration in both wavelength and polarization state, by adding photoresponsive molecular switches into a HPC mesophase. The control experiment with photochromic SP‐**3** highlighted the orthogonality of the approach, allowing for it to be combined to enable additional optical readouts. While this work primarily provides a fundamental basis for the understanding of chiral photomodulation in dopant‐matrix systems in cellulose derivatives, italso showcases the potential of chirality‐controlled structurally colored systems in various applications, including dynamic displays, visual sensors, or security labelling, due to the simple fabrication process combined with the low cost of the materials used.

## Conflict of Interests

The authors declare no conflict of interest.

## Supporting information



Supporting Information

## Data Availability

The data that support the findings of this study are openly available from the University of Cambridge data repository at: https://doi.org/10.17863/CAM.121614.
