# Supplementary material for: Tuning the Circularly Polarized Reflection from Cholesteric Hydroxypropyl Cellulose Using Molecular Photoswitches
Source: Angew Chem Int Ed Engl. 2025 Nov 10;65(1):e20839. doi: 10.1002/anie.202520839 (PMC12759224; doi:10.1002/anie.202520839)
Supplement: Supplementary file 1 — Supporting Information [file ANIE-65-e20839-s001.pdf]

Supporting Information
©Wiley-VCH 2021
69451 Weinheim, Germany

Tuning the Circularly Polarized Reflection from Cholesteric Hydroxypropyl Cellulose Using Molecular Photoswitches

Fathy Hassan,[a, b] Chun Lam Clement Chan,[a] Thomas G. Parton,[a, c] Zhen Wang,[a, c] Qingchen Shen,[a] Ruiting Li,[a, c] Bruno Frka-Petesic, * [a, d] Richard M. Parker,*[a] and Silvia Vignolini*[a, c]
[a]Yusuf Hamied Department of Chemistry, University of Cambridge, Lensfield Road, Cambridge, CB2 1EW, United Kingdom.
[b]Department of Chemistry, Faculty of Science, Tanta University, Tanta, El-Gharbia 31527, Egypt.
[c]Department of Sustainable and Bio-inspired Materials, Max Planck Institute of Colloids and Interfaces, Am Mühlenberg 1, Potsdam 14476, Germany.
[d]International Institute for Sustainability with Knotted Chiral Meta Matter (WPI-SKCM^2), 1-3-1 Kagamiyama, Hiroshima University, Higashi-Hiroshima, Hiroshima, 739-8526, Japan.

*E-mail: sv319@cam.ac.uk ; rmp53@cam.ac.uk ; bf284@cam.ac.uk

The data that support the findings of this study are openly available here: https://doi.org/10.17863/CAM.121614.

Table of Contents

1. Materials S2
2. Synthesis S3
3. Methods S6
4. Photoisomerization and tuning the color of cholesteric HPC using molecular switch **1** S9
5. Photo and thermo-responsive behavior of HPC in DMSO (control experiment) S11
6. Thermo-responsive behavior of HPC-**1** S11
7. The cholesteric pitch change after UV light illumination and after heating for HPC-**1** S12
8. DFT calculation for molecular switch **1** S13
9. Photoisomerization and the effect on cholesteric HPC using molecular switch **3** S13
10. Photoisomerization and controlling the degree of circular polarization using **2** and **2′** S15
11. Photoisomerization effect on polarization state of the reflection in case of HPC-**1** S21
12. Photoisomerization effect on polarization state of the reflection using **2** and **2′** S22
13. Thermo-responsive behavior for full color change from blue to red S24
14. Comparative optical analysis of the thermo- and photo-responsive behaviors S26
15. Photomasking patterns and writing with UV light beam applications for HPC-**3** S29
16. ^{1}H- and ^{13}C-NMR spectra S30
17. Supplementary references S37

1. Materials

Hydroxypropyl cellulose (dry powder, HPC SSL SFP, Lot No NHC-4221S, food grade, M_W = 40,000 g mol^{-1}, as reported by manufacturer) was purchased from Nisso Chemical Europe. Note that the same batch of HPC was used throughout this work to ensure a valid comparison across the prepared samples, since even subtle changes such as average molecular weight, chain polydispersity and substitution levels of hydroxypropyl groups could significantly affect the pitch, reflection color, and switching behavior. 4-(4-Butylphenylazo)phenol (**1**), 4'-pentyl-4-biphenylboronic acid, and [4-[bis(4-methoxyphenyl)amino]phenyl]boronic acid were purchased from Tokyo Chemical Industry UK, Ltd. 1′,3′-Dihydro-1′,3′,3′-trimethyl-6-nitrospiro[2*H*-1-benzopyran-2,2′-(2*H*)-indole] (**3**), (*R*)-1,1′-binaphthyl-2,2′-diamine ((*R*)-BINAM), (*S*)-1,1′-binaphthyl-2,2′-diamine ((*S*)-BINAM), 4-bromoaniline, dimethyl sulfoxide (DMSO), tetrakis(triphenylphosphine) palladium(0) ($Pd(PPh_3)_4$), potassium peroxymonosulfate (OXONE), potassium carbonate (K_2CO_3), magnesium sulfate ($MgSO_4$), acetic acid, tetrahydrofuran (THF), dichloromethane (DCM), deuterated chloroform ($CDCl_3$), 1,4-dioxane (spectroscopic grade), and deuterated 1,4-dioxane were purchased from Sigma-Aldrich. All materials were used as supplied and without further purification. The chemical structures of the molecular switches used in this study are shown in **Scheme S1**.

a Molecular switch **1**

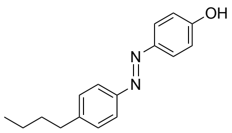

trans-**1**

b Molecular switches **2** and **2′**

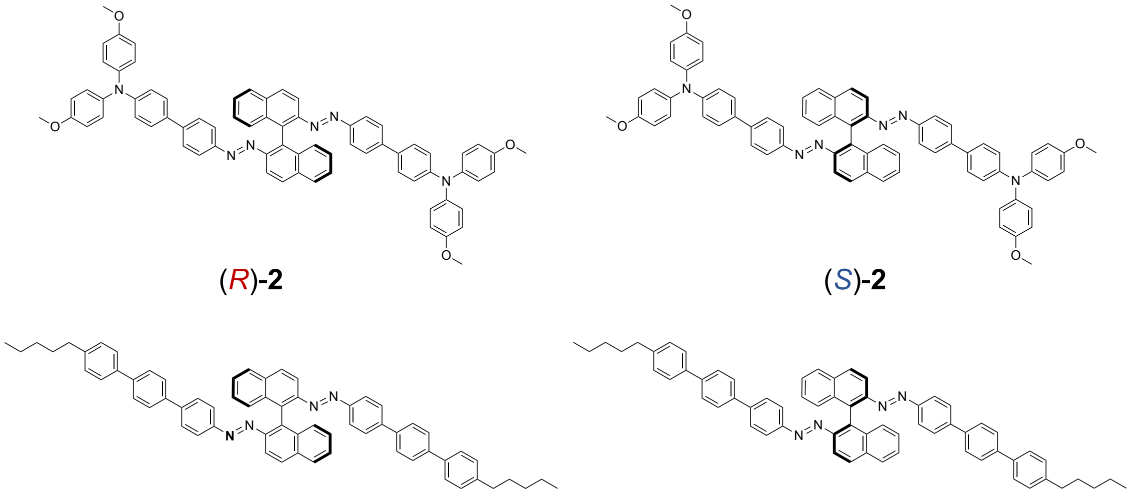

c Molecular switch **3**

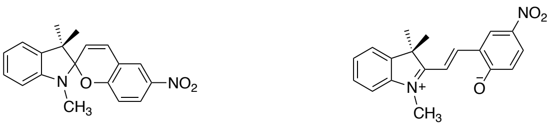

SP-**3** MR-**3**

Scheme S1. Chemical structures of the molecular switches used in this study: (a) azobenzene **1**, (b) chiral binaphthyl-azo derivatives (*R*)-**2**, (*R*)-**2′**, (*S*)-**2**, and (*S*)-**2′**, and (c) spiropyran SP-**3** and merocyanine MR-**3**.

2. Synthesis

The synthetic pathway of the axially chiral photoswitches **2** and **2′** is shown in **Scheme S2**.[S1] First, 1-bromo-4-nitrosobenzene (NOPhBr) was synthesized from 4-bromoaniline by OXONE oxidation in a biphasic system. Then, the obtained nitroso species was reacted with (*R*)-BINAM or (*S*)-BINAM in presence of acetic acid to yield the intermediate (DiBrAzo). The obtained DiBrAzo was reacted with [4-[bis(4-methoxyphenyl)amino]phenyl]boronic acid or 4'-pentyl-4-biphenylboronic acid via a palladium catalyzed Suzuki-Miyaura reaction to yield (*R*)-**2** and (*S*)-**2** or (*R*)-**2′** and (*S*)-**2′**, respectively.

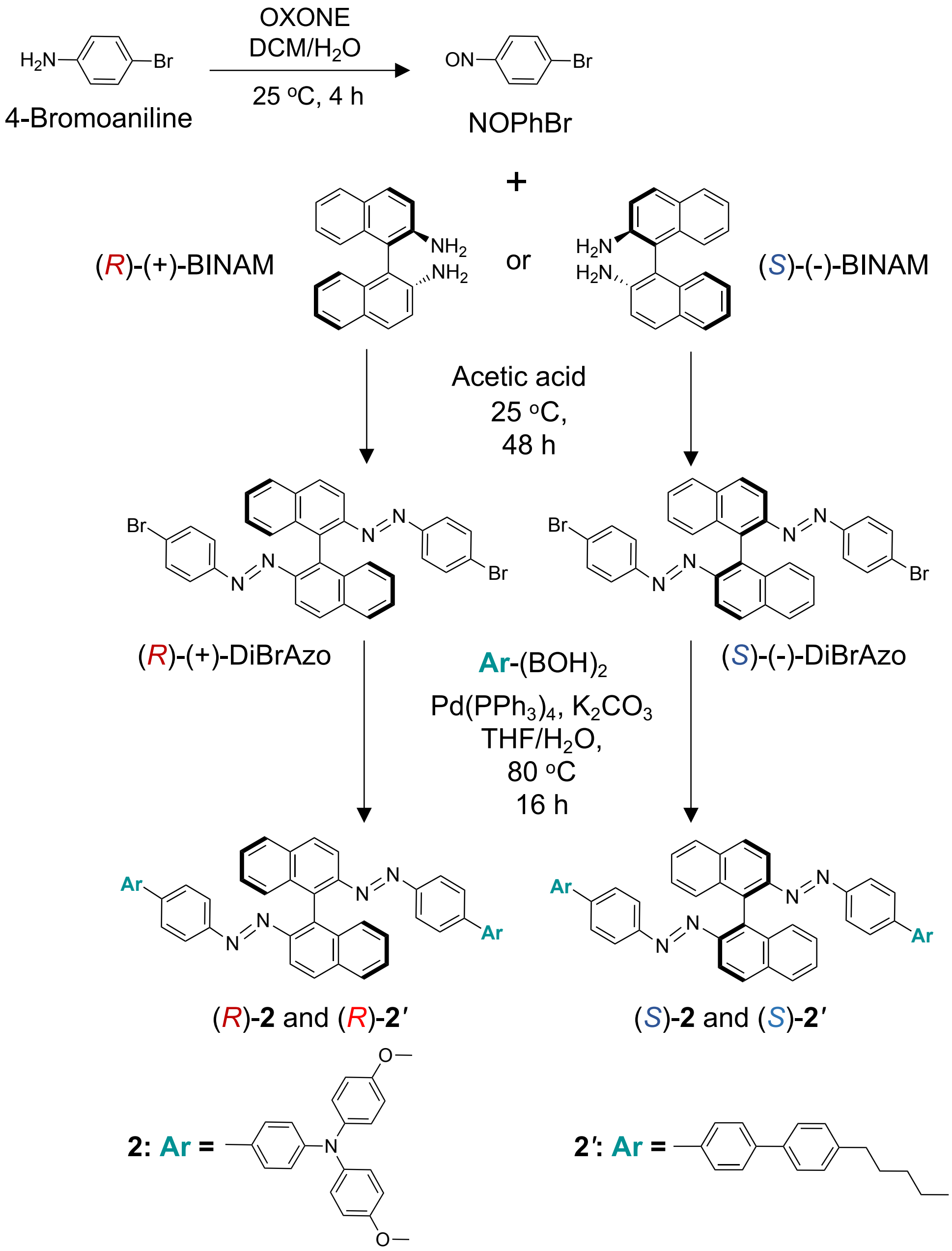

Scheme S2. Synthetic scheme of the axially chiral photoswitches: (*R*)-**2** and (*R*)-**2′** and their enantiomers (*S*)-**2** and (*S*)-**2′**.

Characterization of the synthesized compounds

^{1}H- and ^{13}C-NMR spectra were recorded on a 400 MHz Avance III HD Spectrometer using $CDCl_3$ as a solvent at ambient temperature. Chemical shifts (*δ*) are in ppm with the residual solvent peak as the internal standard. The solvent peak ($CDCl_3$): ^{1}H, 7.26 ppm; ^{13}C, 76.74, 77.05, 77.37 ppm. The coupling constant (*J*) is reported in hertz (Hz). NMR splitting patterns are designated as follows: s, singlet; d, doublet; t, triplet; q: quartet, and m, multiplet. High-resolution mass spectra (HRMS) were recorded on a Waters Xevo G2-S ASAP spectrometer and/or on a Thermo Scientific LTQ Orbitrap XL spectrometer. Column chromatography was carried out on silica gel (230-400 mesh). Analytical thin layer chromatography (TLC) was performed on commercially aluminum backed silica gel (200 micron). Spots on the TLC plates were visualized by exposing to UV light.

NOPhBr. 4-Bromoaniline (5.0 g, 30 mmol) was dissolved in DCM (100 mL). To this solution, OXONE (36.5 g, 60 mmol) dissolved in water (200 mL) was added. The solution mixture was stirred under argon at room temperature until TLC monitoring indicated complete consumption of the starting material (2 h). After separation of the layers, the aqueous layer was extracted with DCM twice. The combined organic layers were washed with 1M HCl, saturated sodium bicarbonate solution, water, and brine, and dried over $MgSO_4$. Removal of the solvent was carried out under reduced pressure at room temperature. The crude product was purified directly by column chromatography on silica gel (eluent: hexane:DCM = 95:5 v/v) yielded 3.7 g (53%) of **NOPhBr** as a green solid which was stored at -5 °C.
^{1}H-NMR ($CDCl_3$, 400 MHz, **Figure S28a**): *δ* = 7.78 (s, 4H, Ar H). ^{13}C-NMR ($CDCl_3$, 400 MHz, **Figure S28b**): *δ* = 122.11 (C_2), 131.69 (C_4), 132.69 (C_3), 163.80 (C_1) ppm. HRMS calcd. for C_6H_5NOBr ($[M+H]^+$): 158.9555, found: 158.9561.

(*R*)-DiBrAzo. (*R*)-BINAM (0.5 g, 1.75 mmol) was dissolved in acetic acid (50 mL). To this solution, **NOPhBr** (0.71 g, 3.9 mmol) dissolved in acetic acid (50 mL) was added. The reaction mixture was stirred for 48 h at 25 °C under argon. The resulting mixture was quenched by distilled water (200 mL), extracted with DCM (300 mL), and dried over $MgSO_4$. The solvent was evaporated under reduced pressure, and the residue was purified by column chromatography on silica gel (eluent: hexane:ethyl acetate = 90:10 v/v) to afford **(*R*)-DiBrAzo** (0.55 g, 51%) as a red powder.
^{1}H-NMR ($CDCl_3$, 400 MHz, **Figure S29a**): *δ* = 7.17–7.19 (d, 4H, *J* = 8.8 Hz), 7.29–7.34 (t, 2H), 7.37–7.39 (d, 4H, *J* = 8.8 Hz), 7.47–7.50 (d, 2H, *J* = 8 Hz), 7.54–7.58 (t, 2H), 8.01–8.03 (d, 2H, *J* = 8.6 Hz), 8.08–8.10 (d, 2H, *J* = 8.6 Hz), 8.15–8.17 (d, 2H, *J* = 8 Hz). ^{13}C-NMR ($CDCl_3$, 100 MHz, **Figure S29b**): *δ* = 114.08, 124.14, 124.92, 126.86, 127.53, 127.88, 128.17, 129.31, 131.99, 134.11, 134.55, 137.62, 148.15, 151.45 ppm. HRMS calcd. for $C_{32}H_{21}Br_2N_4$ ($[M+H]^+$): 619.0133, found: 619.0139.

(*S*)-DiBrAzo. Same procedure for **(*R*)-DiBrAzo** is applied but using (*S*)-BINAM instead of (*R*)-BINAM to afford **(*S*)-DiBrAzo** (0.58 g, 53%) as a red powder.
^{1}H-NMR ($CDCl_3$, 400 MHz, **Figure S30a**): *δ* = 7.18–7.20 (d, 4H, *J* = 8.6 Hz), 7.29–7.38 (m, 6H), 7.49–7.507(m, 4H), 8.00–8.02 (d, 2H, *J* = 8.6 Hz), 8.08–8.10 (d, 2H, *J* = 8.6 Hz), 8.17–8.19 (d, 2H, *J* = 8 Hz). ^{13}C-NMR ($CDCl_3$, 100 MHz, **Figure S30b**): *δ* = 114.09, 123.91, 124.16, 124.92, 126.86, 127.23, 127.90, 128.17, 129.31, 132.02, 134.13, 134.58, 137.60, 148.17, 151.50 ppm. HRMS calcd. for $C_{32}H_{21}Br_2N_4$ ($[M+H]^+$): 619.0133, found: 619.0137.

(*R*)-2. To a solution of **(*R*)-DiBrAzo** (0.2 g, 0.3 mmol, 1.00 equiv) in THF (50 mL), [4-[bis(4-methoxyphenyl)amino]phenyl]boronic acid (0.23 g, 0.66 mmol, 2.2 equiv) was added under argon. To this mixture, a solution of K_2CO_3 (0.18 g, 1.32 mmol, 4.4 equiv) in distilled water (15 ml) was added. After stirring under reduced pressure, $Pd(PPh_3)_2$ (35 mg, 0.03 mmol, 10 mol%) catalyst was added. The

reaction mixture was refluxed for 16 h at 80 °C under argon. TLC analysis showed completion of the reaction. THF was removed under vacuum. The residue was extracted with DCM (300 mL) and water (200 mL). The organic layer was dried over $MgSO_4$ and concentrated under reduced pressure. It was then purified by column chromatography over silica gel (eluent: hexane:ethyl acetate = 70:30 v/v) to afford **(*R*)-2**. (0.19 g, 53%) as a red powder.
^{1}H-NMR ($CDCl_3$, 400 MHz, **Figure S31a**): *δ* = 3.80 (s, 12H), 6.82–6.85 (d, 8H, *J* = 12 Hz), 6.91–6.93 (d, 4H, *J* = 8 Hz), 7.06–7.08 (d, 8H, *J* = 8 Hz), 7.28–7.38 (m, 14H), 7.47–7.53 (q, 4H), 7.99–8.01 (d, 2H, *J* = 8 Hz), 8.07–8.09 (d, 2H, *J* = 8.8 Hz), 8.19–8.21 (d, 2H, *J* = 8 Hz). ^{13}C-NMR ($CDCl_3$, 100 MHz, **Figure S31b**): δ = 55.49, 114.39, 114.73, 120.25, 123.34, 126.51, 126.78, 126.80, 127.17, 127.43, 127.92, 129.09, 129.11, 131.60, 134.29, 134.42, 137.30, 140.62, 142.78, 148.49, 148.57, 151.44, 156.03 ppm. HRMS calcd. for $C_{72}H_{56}N_6O_4$ ($[M]^+$): 1068.4358, found: 1068.4323.

(*S*)-2. Same procedure for **(*R*)-2** is applied but using **(*S*)-DiBrAzo** instead of **(*R*)-DiBrAzo** to afford **(*S*)-3** (0.21 g, 54%) as a red powder.
^{1}H-NMR ($CDCl_3$, 400 MHz, **Figure S32a**): *δ* = 3.82 (s, 12H), 6.85–6.87 (d, 8H, *J* = 12 Hz), 6.93–6.95 (d, 4H, *J* = 8 Hz), 7.08–7.10 (d, 8H, *J* = 8 Hz), 7.32–7.40 (m, 14H), 7.47–7.53 (q, 4H), 7.99–8.01 (d, 2H, *J* = 8 Hz), 8.07–8.09 (d, 2H, *J* = 8.8 Hz), 8.19–8.21 (d, 2H, *J* = 8 Hz). ^{13}C-NMR ($CDCl_3$, 100 MHz, **Figure S32b**): δ = 55.51, 114.38, 114.73, 120.24, 123.36, 126.53, 126.69, 126.80, 127.19, 127.44, 127.92, 128.11, 129.11, 131.60, 134.29, 134.43, 137.31, 140.62, 142.79, 148.48, 148.57, 151.44, 156.03 ppm. HRMS calcd. for $C_{72}H_{56}N_6O_4$ ($[M]^+$): 1068.4358, found: 1068.4326.

(*R*)-2′. To a solution of compound **(*R*)-DiBrAzo** (0.2 g, 0.3 mmol) in THF (50 mL), 4'-pentyl-4-biphenylboronic acid (0.18 g, 0.66 mmol, 2.2 equiv) was added under argon. To this mixture, a solution of K_2CO_3 (0.18 g, 1.32 mmol, 4.4 equiv) in distilled water (15 ml) were added. After stirring under reduced pressure, $Pd(PPh_3)_2$ catalyst was added (35 mg, 0.03 mmol, 10 mol %). The reaction mixture was refluxed for 16 h at 80 °C under argon. TLC analysis showed completion of the reaction. THF was removed under vacuum. The residue was extracted with DCM (300 mL) and water (200 mL). The organic layer was dried over $MgSO_4$ and concentrated under reduced pressure. It was then purified by column chromatography over silica gel (eluent: hexane:DCM = 70:30 v/v) to afford **(*R*)-2′** (0.15 g, 51%) as a red powder.
^{1}H-NMR ($CDCl_3$, 400 MHz, **Figure S33a**): *δ* = 0.92–0.96 (t, 6H, *J* = 8 Hz), 1.36–1.41 (m, 8H), 1.65–1.72 (m, 4H), 2.65–2.69 (t, 4H, *J* = 8.8 Hz), 7.27–7.35 (m, 6H), 7.43–7.45 (d, 2H, *J* = 8.8 Hz), 7.52–7.58 (m, 12H), 7.59–7.65 (m, 8H), 8.03–8.05 (d, 4H, *J* = 8.8 Hz), 8.11–8.13 (d, 2H, *J* = 8 Hz), 8.24–8.26 (d, 2H, *J* = 8.8 Hz). ^{13}C-NMR ($CDCl_3$, 100 MHz, **Figure S33b**): δ = 14.07, 22.59, 31.20, 31.59, 35.63, 114.36, 123.37, 126.78, 126.85, 127.32, 127.97, 128.16, 128.91, 129.20, 134.31, 134.54, 137.52, 137.81, 138.71, 140.55, 142.38, 142.72, 148.49, 152.04 ppm. HRMS calcd. for $C_{66}H_{59}N_4$ ($[M+H]^+$): 907.4740, found: 907.4747.

(*S*)-2′. Same procedure for **(*R*)-2′** is applied but using **(*S*)-DiBrAzo** instead of **(*R*)-DiBrAzo** to afford **((*S*)-2′** (0.12 g, 48%) as a red powder.
^{1}H-NMR ($CDCl_3$, 400 MHz, **Figure S34a**): *δ* = 0.91–0.95 (t, 6H, *J* = 8 Hz), 1.36–1.39 (m, 8H), 1.64–1.71 (m, 4H), 2.65–2.69 (t, 4H, *J* = 8.8 Hz), 7.27–7.28 (d, 4H), 7.32-7.36 (t, 2H, *J* = 8 Hz), 7.43–7.45 (d, 4H, *J* = 8.8 Hz), 7.52–7.58 (m, 12H), 7.59–7.65 (m, 8H), 8.03–8.05 (d, 2H, *J* = 8.8 Hz), 8.11–8.13 (d, 2H, *J* = 8 Hz), 8.24–8.26 (d, 2H, *J* = 8.8 Hz). ^{13}C-NMR ($CDCl_3$, 100 MHz, **Figure S34b**): δ = 14.07, 22.59, 31.20, 31.59, 35.63, 114.36, 123.37, 126.78, 126.85, 127.32, 127.97, 128.16, 128.91, 129.20, 134.31, 134.54, 137.52, 137.81, 138.71, 140.55, 142.38, 142.72, 148.49, 152.04 ppm. HRMS calcd. for $C_{66}H_{59}N_4$ ($[M+H]^+$): 907.4740, found: 907. 4717.

3. Methods

Photoresponsive behavior

The photoresponsive behavior of molecular switches **1-3** was studied using a Varian Cary 400 UV/Vis spectrophotometer using a cuvette with 1 cm path length at room temperature (~20-25 °C) in 1,4-dioxane and in DMSO with the PSSs being the same in both solvents. Solutions were prepared at a concentration of 5 µM. The data in 1,4-dioxane are presented to highlight the exciton couplets around 220 nm, which originate from the ^1B_b transitions of the 1,1'-binaphthyl. These couplets are not visible in DMSO due to overlap with its absorption.

Chiroptical switching behavior

The chiroptical switching behavior of chiral molecular switches **2** and **2′** was studied using an Applied Photophysics Chirascan circular dichroism (CD) spectrophotometer in 1,4-dioxane and in DMSO. Solutions were prepared at a concentration of 0.5 mM.

trans:cis ratio in the photostationary state

^{1}H-NMR spectra of compounds **1**, **2**, and **2′** were performed in deuterated 1,4-dioxane after 1 h of light illumination at the desired wavelength and compared with the ^{1}H-NMR spectrum of their initial state.

DFT calculation

Computational chemical predictions were performed with the Gaussian 09 program, Revision E01. The optimized geometry and the electron density potential map of the isomers were based on the density functional theory (DFT) using the DFT B3LYP/6-31G basis.

Light illumination

Samples were irradiated at 365 nm (40 mW/cm^2) using a UV LED (1.2 W power output, LED Engin) and neutral density filter (NE10A, Thorlabs) attached to a heat sink (ATSEU-077B-C2-R0, Advanced Thermal Solutions). Samples were irradiated at 405 nm (25 mW/cm^2) using a mounted blue LED (M405LP1, Thorlabs), and similarly at 455 nm (20 mW/cm^2) using another mounted blue LED (M455L4, Thorlabs). Samples were irradiated at 520 nm (5 mW/cm^2) using a broadband xenon light source (LAX-C100 W, Asahi Spectra) filtered through a 520 nm filter.

Light intensity measurement

The light intensity at the sample surface was estimated using a photodiode (S120C, Thorlabs) for the visible light sources and a thermal power sensor (S175C, Thorlabs) for the UV light source.

Preparation of HPC-photoswitch mesophases

DMSO (2.8 g) and molecular switches (**1-3,** 0.01 g) were mixed with HPC (7.2 g) using a Thinky ARE-250 planetary centrifuge mixer (1600 rpm, 2 min, 5 repeats) to produce an HPC mesophase at 72 wt.% containing the photoswitches (**1-3**) at 0.1 wt.%. To remove bubbles, the mixture was degassed by high-speed centrifugation (7000 rpm in Thermo Scientific, Heraeus Multifuge X1R Centrifuge) for 30 min.

Fabrication of the characterization cell

The encapsulated films were prepared by spreading the HPC mesophase on a microscope glass slide (75 x 52 mm). A second microscope slide was then placed on top and fixed with a strip of adhesive tape.

Letters were engraved in the PMMA substrate using a 3D laser cutter. All samples were kept overnight before measurement to obtain a uniform HPC mesophase.

Note that in such confinement the HPC mesophase spontaneously organizes into a polydomain cholesteric structure throughout the thickness of the cell, but with aligned regions near the glass surface oriented with the helical axis normal to the interface, due to a degenerated planar anchoring at the liquid/glass interface.[S2]-[S7]

Film thickness measurements

The film thickness was measured from an optical micrograph obtained using a VHX-E500 Keyence Digital Microscope (**Figure S1**).

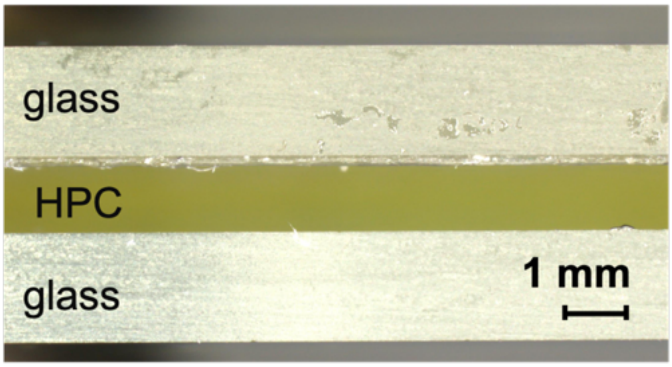

Figure S1. Cross-sectional view of an encapsulated HPC mesophase between glass plates, as imaged by a Keyence digital microscope.

Polarized optical microscopy

Optical microscopy (OM) was performed using a Zeiss Axio microscope and a 10x objective (Zeiss EC Epiplan-Apochromat 10x/0.3 BD DIC M27). Images acquired using a CMOS camera (Pixelink P-LD725C-UT). The experimental configurations for reflection and transmission microscopy during sample irradiation are shown in **Figure S2**. To record reflectance spectra, a beamsplitter was inserted into the light path and the outgoing light was coupled into a spectrometer (Avantes AvaSpec-HS2048) by a 600 µm core optical fiber (Thorlabs FC-UV600) mounted confocal to the imaging plane. Reflection spectra were normalized to a white diffusive reflectance standard (Labsphere SRS-99-010). For polarized optical microscopy (POM), an analyzer composed of a linear polarizer and a quarter-wave plate with its slow axis oriented either at +45° or −45° was inserted into the light path after the objective to select either the RCP or LCP light collected from the sample (**Figure S2**).

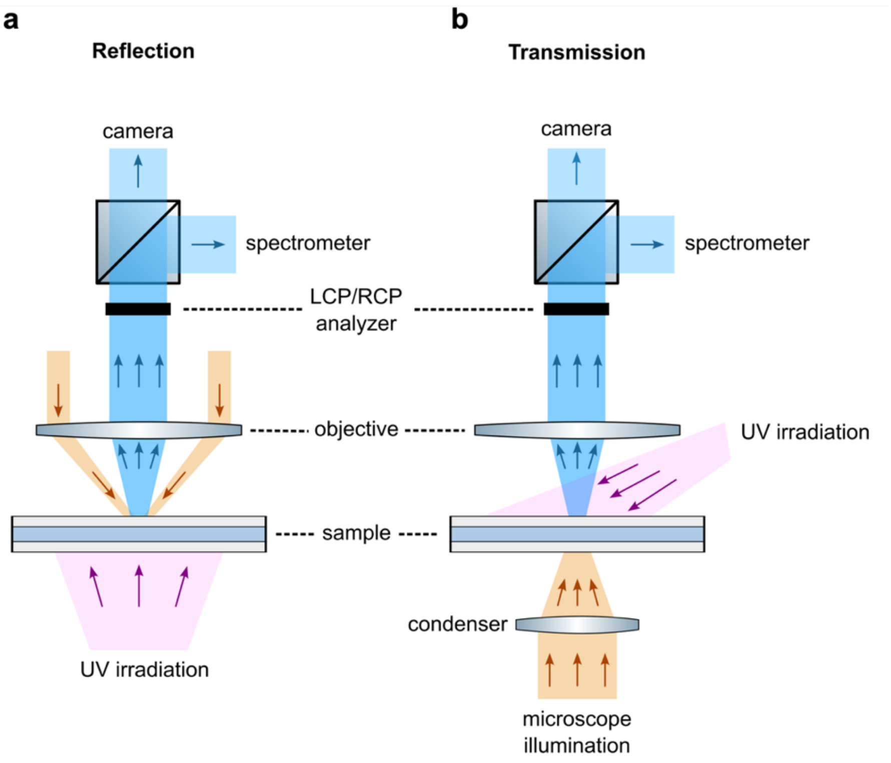

Figure S2. Schematic of the optical microscope setup for POM in reflection (a) and transmission (b) modes with simultaneous UV irradiation.

Temperature measurements

Encapsulated HPC films were heated using a temperature-controlled microscope stage (Linkam Scientific Instruments, THMS600 and TMS-91 controller). The internal temperature was monitored using a Farnell thermocouple wire Type T, 1 M 40AWG attached to Keysight DAQ970A Data Acquisition System (**Figure S3** and **Figure S4**).

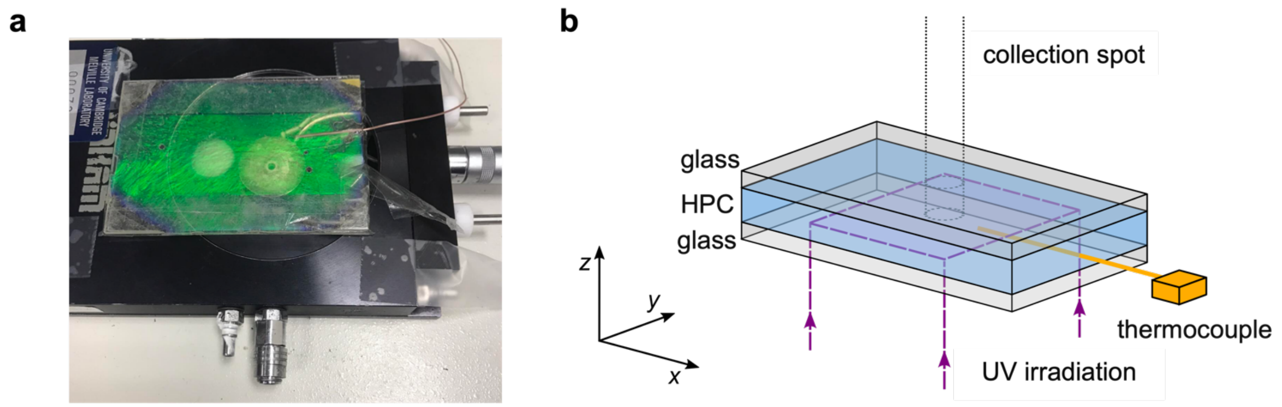

Figure S3. (a) Photograph of the setup for measuring the temperature inside a cholesteric solution of HPC (72 wt.% in DMSO) containing 0.1 wt.% of photoswitch **1**, using thermocouple wire upon heating on the hotplate. (b) Schematic illustration for the setup for measuring the actual temperature inside the HPC mesophase during the light illumination under the microscope using thermocouple wire.

Refractive index

The refractive index n = 1.486 for the mesophase of HPC (72 wt.%) in DMSO was calculated according to the previous report with respect to the volume fractions:[S8]

$$n = \sqrt{n_{\mathrm{HPC}}^2 \Phi_{\mathrm{HPC}} + n_{\mathrm{DMSO}}^2 (1 - \Phi_{\mathrm{HPC}})} \tag{S1}$$

The refractive indices of HPC and DMSO are taken as n_{HPC} = 1.490 and n_{DMSO} = 1.479. The volume fraction of HPC, Φ_{HPC}, was defined as $\Phi_{\mathrm{HPC}} = \rho_{\mathrm{DMSO}} c_{\mathrm{HPC}} / (\rho_{\mathrm{DMSO}} c_{\mathrm{HPC}} + \rho_{\mathrm{HPC}} c_{\mathrm{DMSO}})$, where $c_{\mathrm{HPC}} = 0.72$ is the weight fraction of HPC in DMSO, and the volume masses (densities) of HPC and DMSO were taken as ρ_{HPC} = 1.4 g/cm^3 and ρ_{DMSO} = 1.1 g/cm^3, respectively.

4. Photoisomerization and tuning the color of cholesteric HPC using molecular switch 1

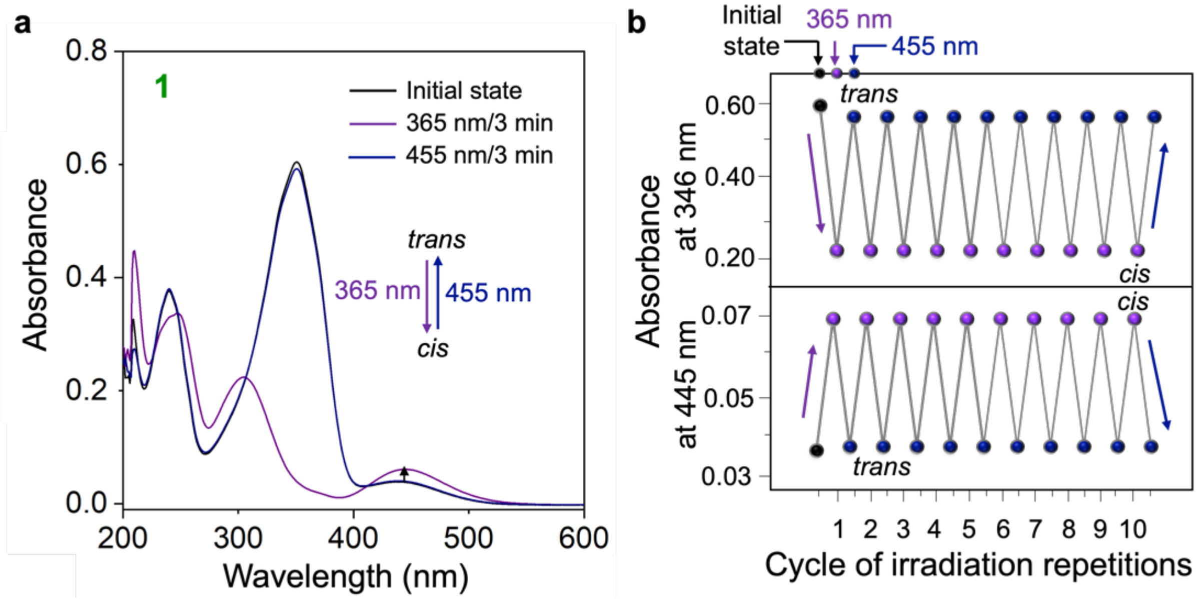

Figure S4. (a) UV-Vis absorption spectra of photoswitch **1** (5 μM) in 1,4-dioxane after light illumination for 3 min. (b) Photoswitchable behavior of the absorbance at monitoring wavelengths of 346 and 445 nm for 10 irradiation cycles.

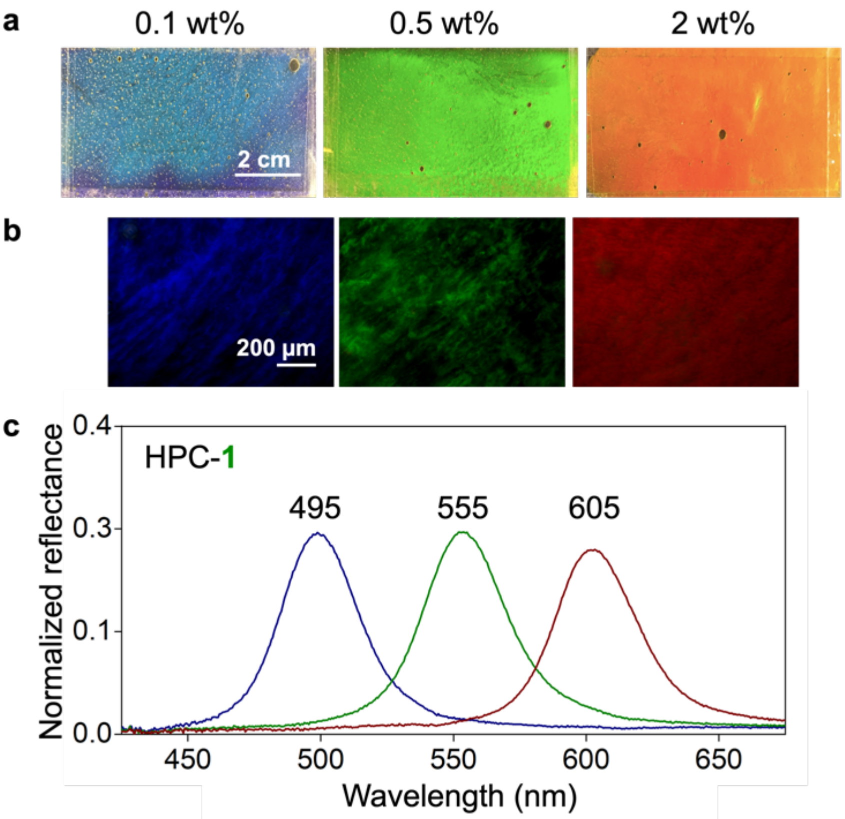

Figure S5. Blue, green, and red HPC mesophases (72 wt.% in DMSO) containing different concentrations of **1**. (a) Photographs of the films. (b) Optical microscopy (OM) images. (c) Reflection spectra (with respective peak wavelengths indicated in nm).

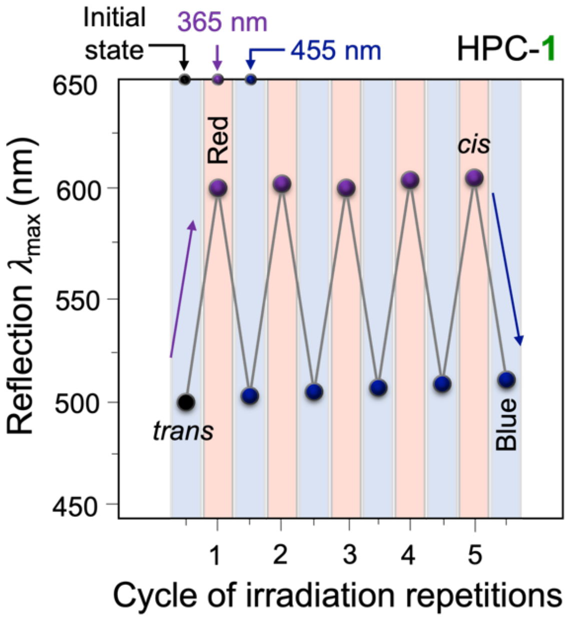

Figure S6. Photoswitchable behavior of an HPC mesophase (72 wt.% in DMSO) containing 0.1 wt.% of photoswitch **1**, showing the color change from blue to red over 5 cycles of alternating irradiation (UV illumination: 40 min, 365 nm, 40 mW cm^{-2}; visible illumination: 30 min, 455 nm, 20 mW cm^{-2}).

5. Photo and thermo-responsive behavior of HPC in DMSO (control experiment)

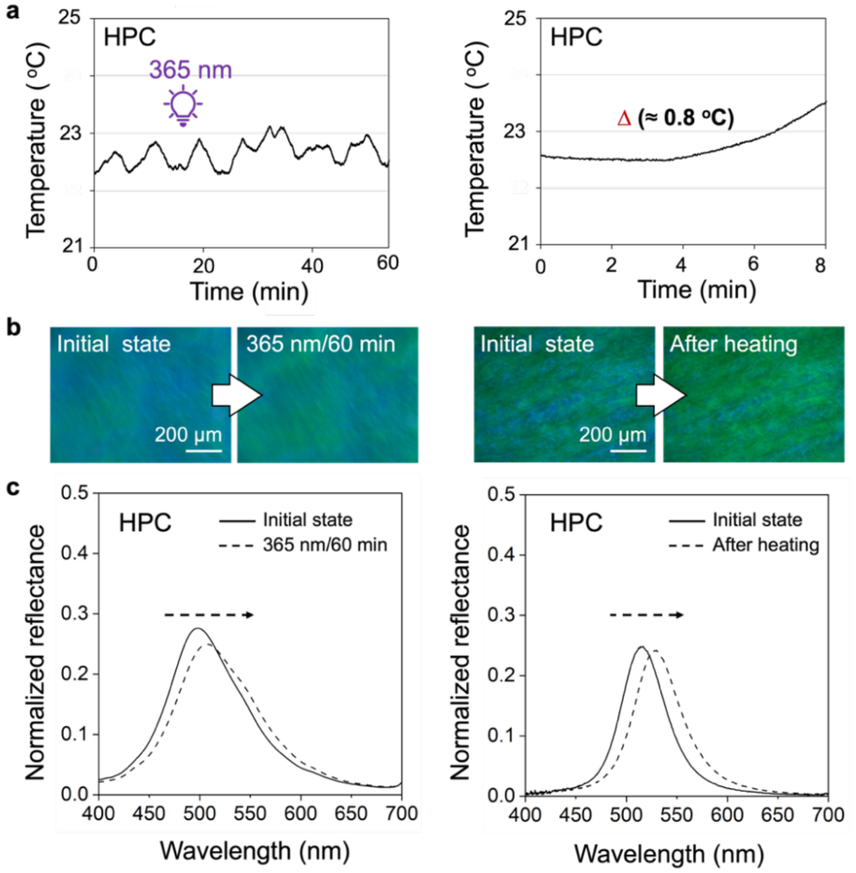

Figure S7. The thermo-responsive behavior of HPC mesophase (72 wt.% in DMSO) in the3 absence of a photoswitch. (a) The monitored temperatures upon UV light illumination starting at room temperature (left) and upon heating to reach the same temperature increase. (b) OM images before and after UV exposure (left) or heating (right). (c) The corresponding change in reflection spectra before and after UV exposure (left) or heating (right).

6. Thermo-responsive behavior of HPC-1

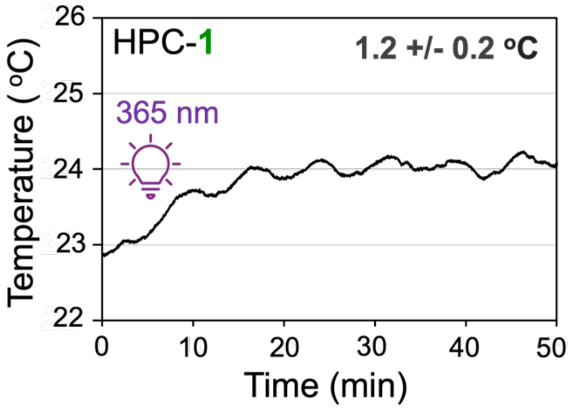

Figure S8. The monitored temperature with illumination under UV light at 365 nm for HPC mesophase (72 wt.% in DMSO) containing 0.1 wt.% of photoswitch **1**.

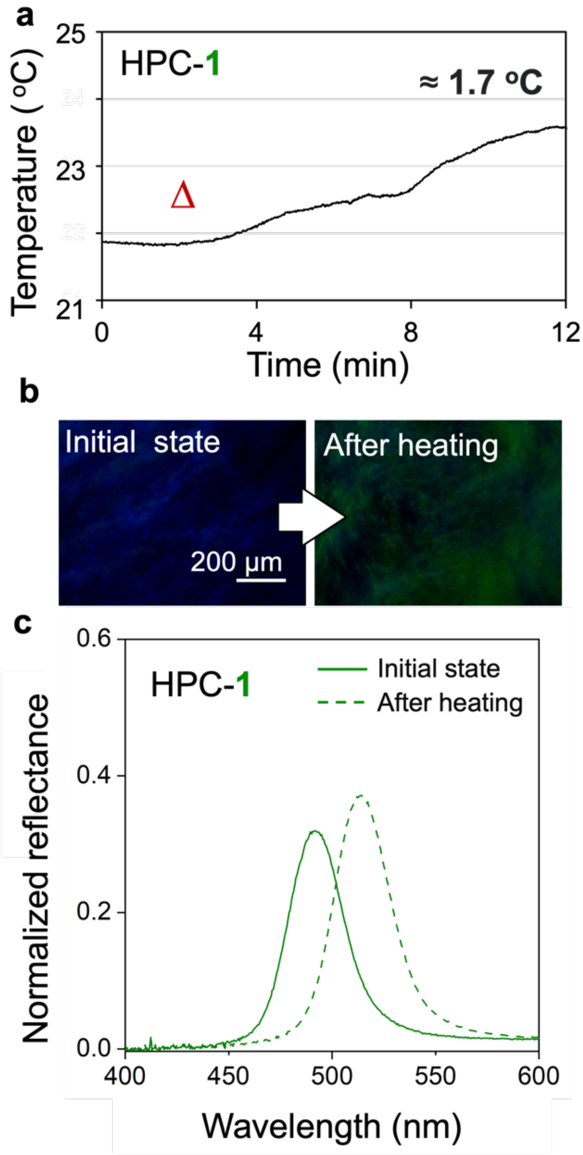

Figure S9. Thermoresponsive behavior of an HPC mesophase (72 wt.% in DMSO) containing 0.1 wt.% of photoswitch **1**. (a) The monitored temperature upon heating for 1.7 °C, comparable to the temperature raise with UV light exposure. (b) OM images for the color change from blue to green after heating. (c) The corresponding change in reflection spectra.

7. The cholesteric pitch change after UV light illumination and after heating for HPC-1

Table S1. Summary for the cholesteric pitch change after UV light illumination and after heating for a comparable temperature range, for an HPC mesophase (72 wt.% in DMSO) containing 0.1 wt.% of photoswitch **1**.

	Pitch, *p* (nm)	
Upon light irradiation starting at RT	Initial state	PSS_{365nm}
	**328 nm**	**405 nm**
Upon controlled temperature rise	21.9 °C	23.6 °C
	**329 nm**	**345 nm**

8. DFT calculation for molecular switch 1

Table S2. Computational chemical predictions for the trans and cis isomers of **1**.[a]

	trans-**1**	*cis*-**1**
Geometry	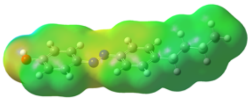	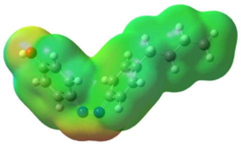
Dipole moment (Debye)	1.66	3.53
C4–C4' distance (Å)[b]	9.14	6.82
CNNC Dihedral angle	180°	11°
Max interatomic distance (Å)	16.7	13.9 (-16.7%)
Molecule length (Å)[c]	17.7	14.9 (-15.8%)
Convex hull surface, S_{ch} ($Å^2$)[c]	313.2	314.4 (+0.4%)
Convex hull volume, V_{ch} ($Å^3$)[c]	343.3	412.3 (+20.1%)
Convex hull isoperimetric quotient[d], $36\pi\ V_{ch}^2 / S_{ch}^3$	0.434	0.619 (+42.5%)
Bare volume, V_b ($Å^3$)[c]	225.1	225.6 (+0.2%)
Compacity V_b / V_{ch}	0.656	0.547 (-16.5%)

[a]: using DFT B3LYP/6-31G basis. [b]: Length between 4- and 4'-carbons in azobenzene. [c]: convex hull shape estimated from an adaptation of the Matlab script 'molecule3D.m' (version 1.2) from André Ludwig (ETHZ), with atomic diameter values set by the 'superlarge' setting (See Open Data for full access).

9. Photoisomerization and the effect on cholesteric HPC using molecular switch 3

Table S3. Computational chemical predictions for SP-**3** and MR-**3**.[a]

	SP-**3**	MR-**3**
Geometry	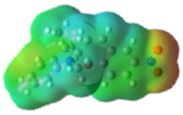	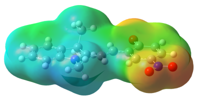
Dipole moment (Debye)	6.41	11.93
N–O distance (Å)[b]	2.39	6.37
CCCC Dihedral angle	118°	180°
Max interatomic distance (Å)	12.4	13.7 (+10.5%)
Molecule length (Å)[d]	13.4	14.7 (+9.8%)
Convex hull surface, S_{ch} ($Å^2$)[c]	317.1	332.3 (+4.8%)
Convex hull volume, V_{ch} ($Å^3$)[c]	440.9	435.9 (-1.1%)
Convex hull isoperimetric quotient[d], $36\pi\ V_{ch}^2 / S_{ch}^3$	0.689	0.586 (-15.0%)
Bare volume, V_b ($Å^3$)[c]	262.7	265.3 (+1%)
Compacity V_b / V_{ch}	0.596	0.609 (+2.2%)

[a]: using DFT B3LYP/6-31G basis. [b]: Length between N atom (indoline) and the O atom (pyran). [c]: convex hull shape estimated from an adaptation of the Matlab script 'molecule3D.m' (version 1.2) from André Ludwig (ETHZ), with atomic diameter values set by the 'superlarge' setting (See Open Data for full access).

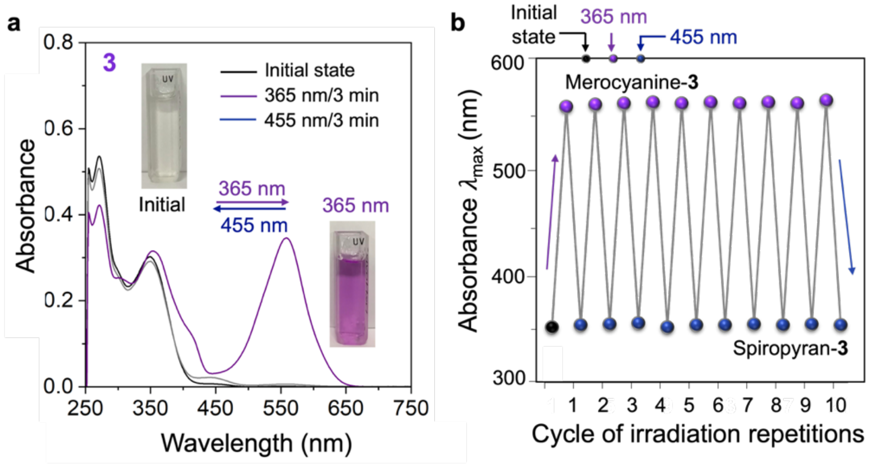

Figure S10. (a) UV-Vis absorption spectra of the photoswitch **3** (5 μM) in 1,4-dioxane after light illumination. Inset: solution color change. (b) Photoswitchable behavior of the absorbance maximum wavelength at 350 nm and 555 nm over 10 cycles of alternating irradiation.

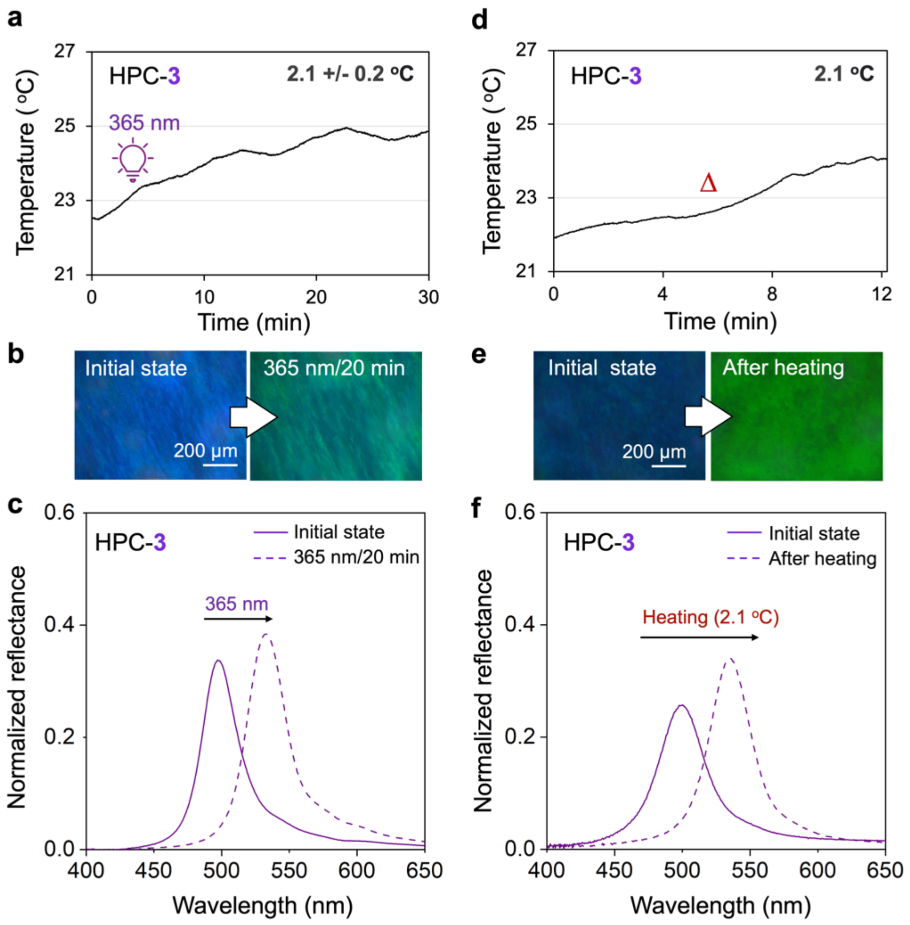

Figure S11. Photoresponsive and thermoresponsive behavior of an HPC mesophase (72 wt.% in DMSO) containing 0.1 wt.% of photoswitch **3**. (a) The monitored temperature with illumination under UV light at 365 nm. (b) OM in reflection before and after irradiation, (c) corresponding reflection spectra. (d) The monitored temperature upon heating for 2.1 °C, comparable to the temperature raised from UV light. (e) OM images for the color change from blue to green after heating. (f) The corresponding change in reflection spectra.

10. Photoisomerization and controlling the degree of circular polarization using 2 and 2′

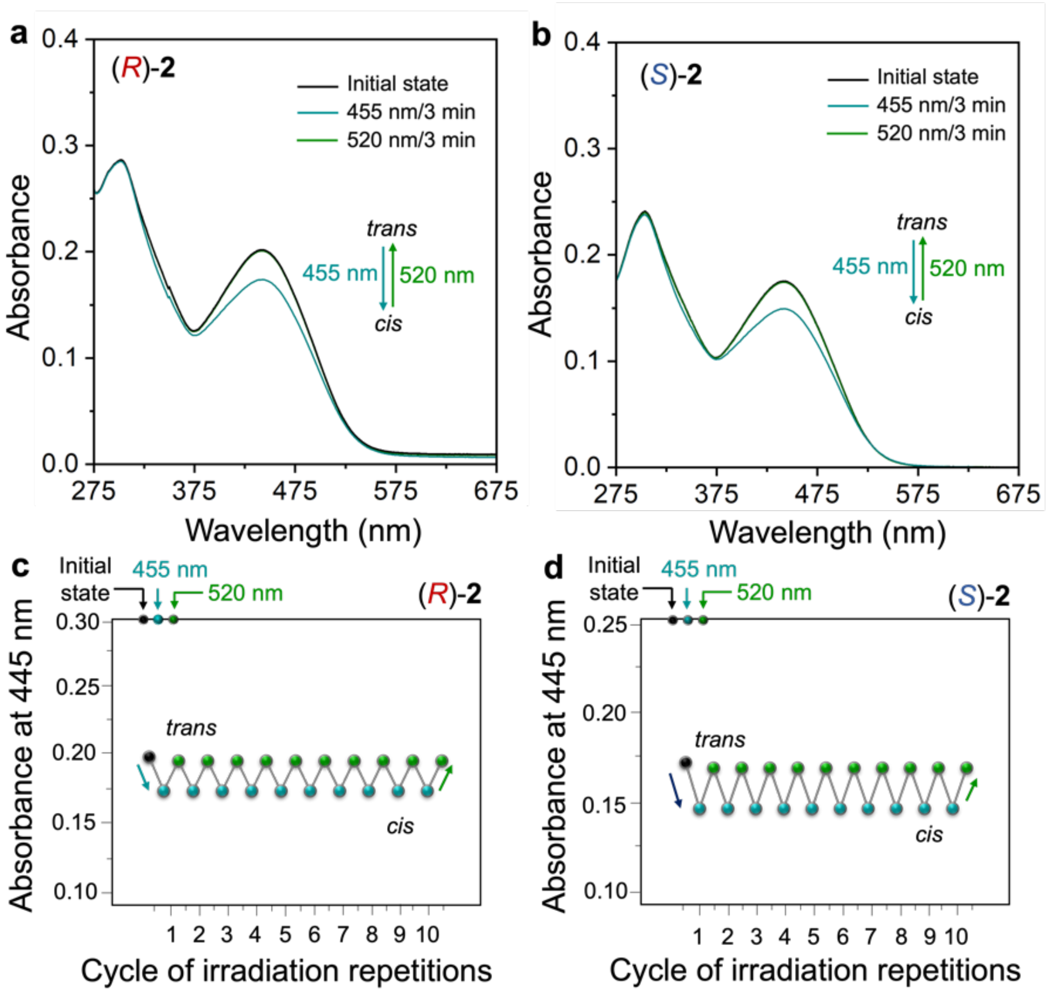

Figure S12. Photoisomerization of chiral photoswitches, (*R*)-**2** and its enantiomer (*S*)-**2** (5 μM), in 1,4-dioxane. (a) UV-Vis absorption spectra of (*R*)-**2** and (b) (*S*)-**2** (the region in the range of 440–550 nm of the spectra is magnified). (c) Photoswitchable behavior of the absorbance of the photoswitch (*R*)-**2** (monitoring wavelength: 445 nm) after repetitions of 10 illumination cycles and (d) corresponding behavior for the photoswitch (*S*)-**2**. (light illumination: 5 min)

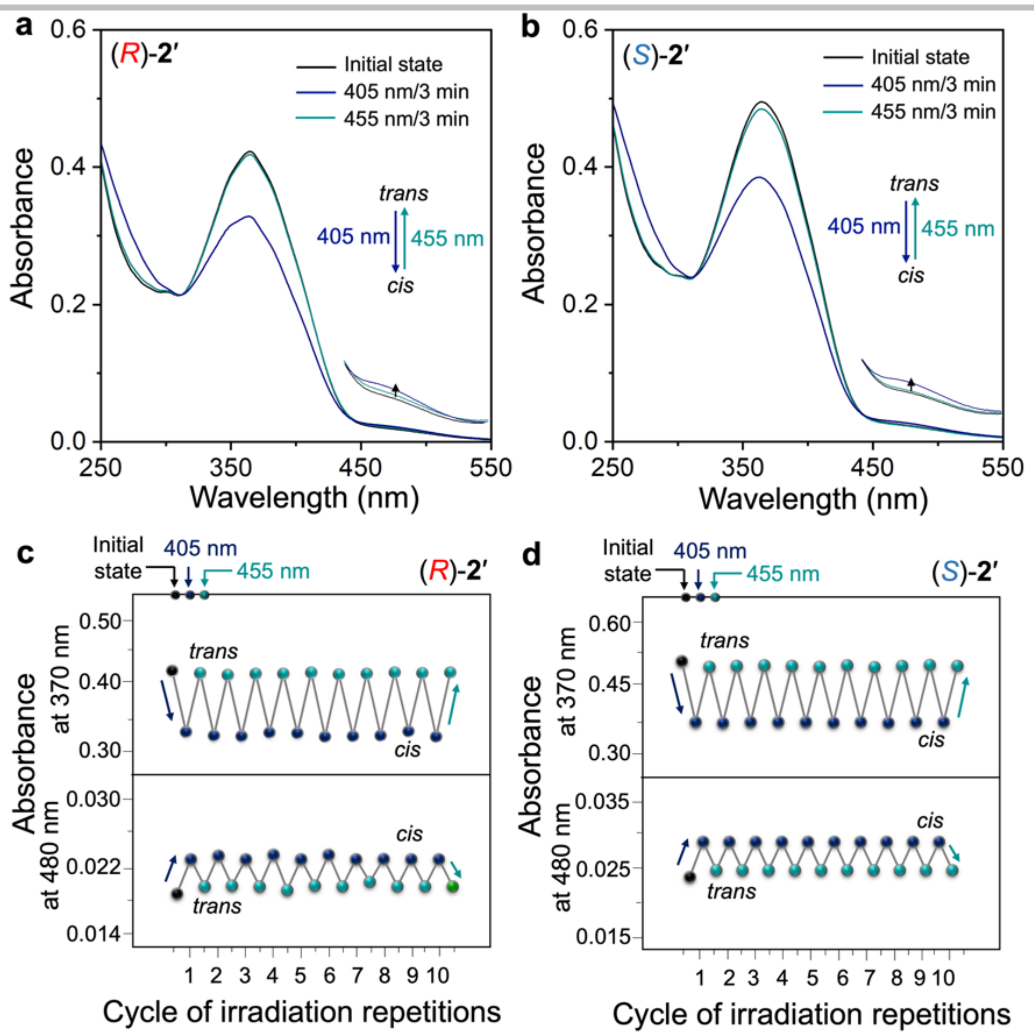

Figure S13. Photoisomerization of chiral photoswitches, (*R*)-**2′** and its enantiomer (*S*)-**2′** (5 μM), in 1,4-dioxane. (a) UV-Vis absorption spectra of (*R*)-**2′** and (b) (*S*)-**2′** (the region in the range of 440–550 nm of the spectra is magnified). (c) Photoswitchable behavior of the absorbance of the photoswitch (*R*)-**2′** (monitoring wavelengths: 370 and 480 nm) after repetitions of 10 illumination cycles and (d) corresponding behavior for the photoswitch (*S*)-**2′**. (light illumination: 5 min)

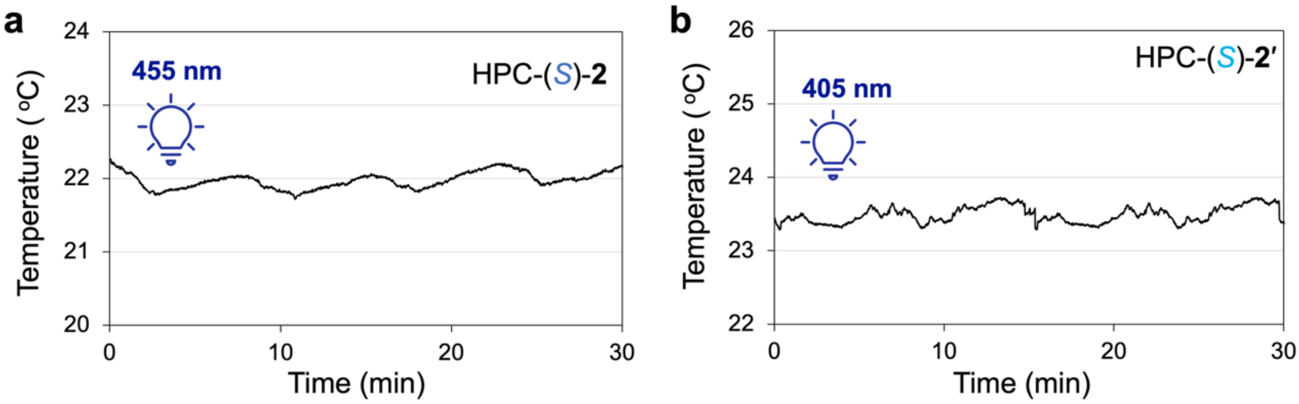

Figure S14. The monitored change in temperature with illumination under visible light in HPC mesophase (72 wt.% in DMSO) with (a) (*S*)-**2** (0.1 wt.%) at 455 nm and (b) (*S*)-**2′** (0.1 wt.%) at 405 nm.

Table S4. Computational chemical predictions for the *trans* and *cis*-isomers of (*S*)-**2**.[a]

	trans-(*S*)-**2**	*cis*-(*S*)-**2**
Geometry	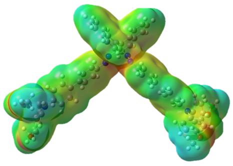	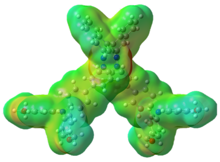
Dipole moment (Debye)	0.77	0.07
C4–C4' distance (Å)[b]	9.16	6.73
CNNC dihedral angle	180°	13°
CCCC dihedral angle	89°	74°
Max interatomic distance (Å)	28.3	26.0 (-8.1%)
Molecule length (Å)[c]	29.3	27.0 (-7.8%)
Convex hull surface, S_{ch} (Å^2)[c]	1468	1402 (-4.5%)
Convex hull volume, V_{ch} (Å^3)[c]	4194	3936 (-6.2%)
Convex hull isoperimetric quotient[d], $36\pi V_{ch}^2 / S_{ch}^3$	0.629	0.636 (+1.2)
Bare volume, V_b (Å^3)[c]	869.0	870.4 (+0.2%)
Compacity V_b / V_{ch}	0.207	0.221 (6.7%)

[a]: using DFT B3LYP/6-31G basis. [b]: Length between 4- and 4'-carbons in azo-binaphthyl. [c]: convex hull shape estimated from an adaptation of the Matlab script 'molecule3D.m' (version 1.2) from André Ludwig (ETHZ), with atomic diameter values set by the 'superlarge' setting (See Open Data for full access).

Table S5. Computational chemical predictions for the *trans* and *cis*-isomers of (*S*)-**2'**.[a]

	trans-(*S*)-**2'**	*cis*-(*S*)-**2'**
Geometry	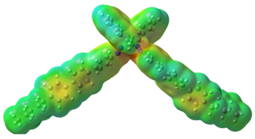	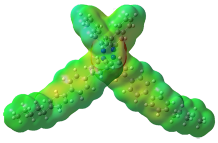
Dipole moment (Debye)	0.77	0.03
C4–C4' distance (Å)[b]	9.15	6.72
CNNC dihedral angle	180°	13°
CCCC dihedral angle	92°	73°
Max interatomic distance (Å)	35.8	35.0 (-2.3%)
Molecule length (Å)[c]	36.8	36.0 (-2.2%)
Convex hull surface, S_{ch} (Å^2)[c]	1390	1290 (-7.2%)
Convex hull volume, V_{ch} (Å^3)[c]	3332	2833 (-15.0%)
Convex hull isoperimetric quotient[d], $36\pi V_{ch}^2 / S_{ch}^3$	0.467	0.423 (-9.5%)
Bare volume, V_b (Å^3)[c]	782.1	782.8 (0.1%)
Compacity V_b / V_{ch}	0.234	0.276 (17.7%)

[a]: using DFT B3LYP/6-31G basis. [b]: Length between 4- and 4'-carbons in azo-binaphthyl. [c]: convex hull shape estimated from an adaptation of the Matlab script 'molecule3D.m' (version 1.2) from André Ludwig (ETHZ), with atomic diameter values set by the 'superlarge' setting (See Open Data for full access).

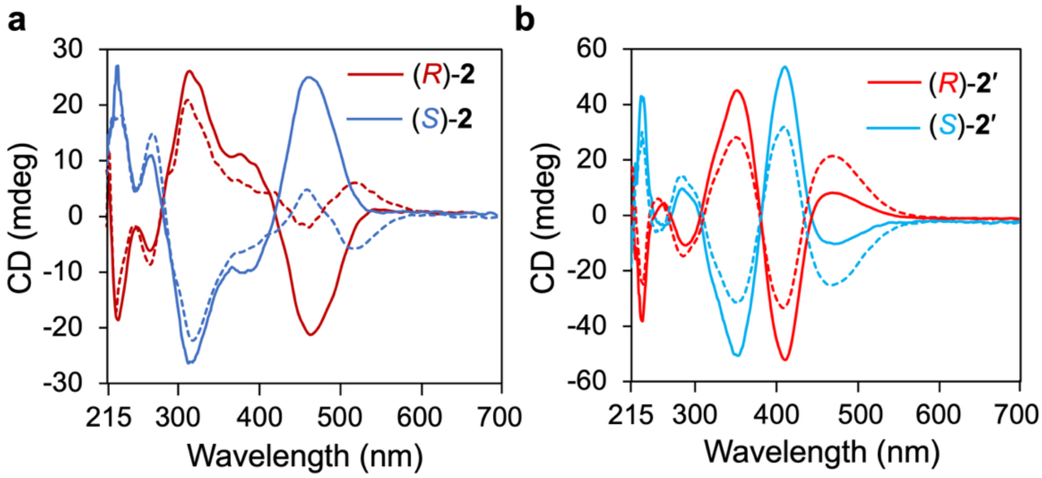

Figure S15. CD spectra of the (*R*)- and (*S*)-enantiomer for both **2** (a) and **2′** (b) in 1,4-dioxane (0.5 mM). Dotted lines for the PSSs after light illumination at 455 nm for (*R*)- and (*S*)- of **2** and at 405 nm for (*R*)- and (*S*)- of **2′**.

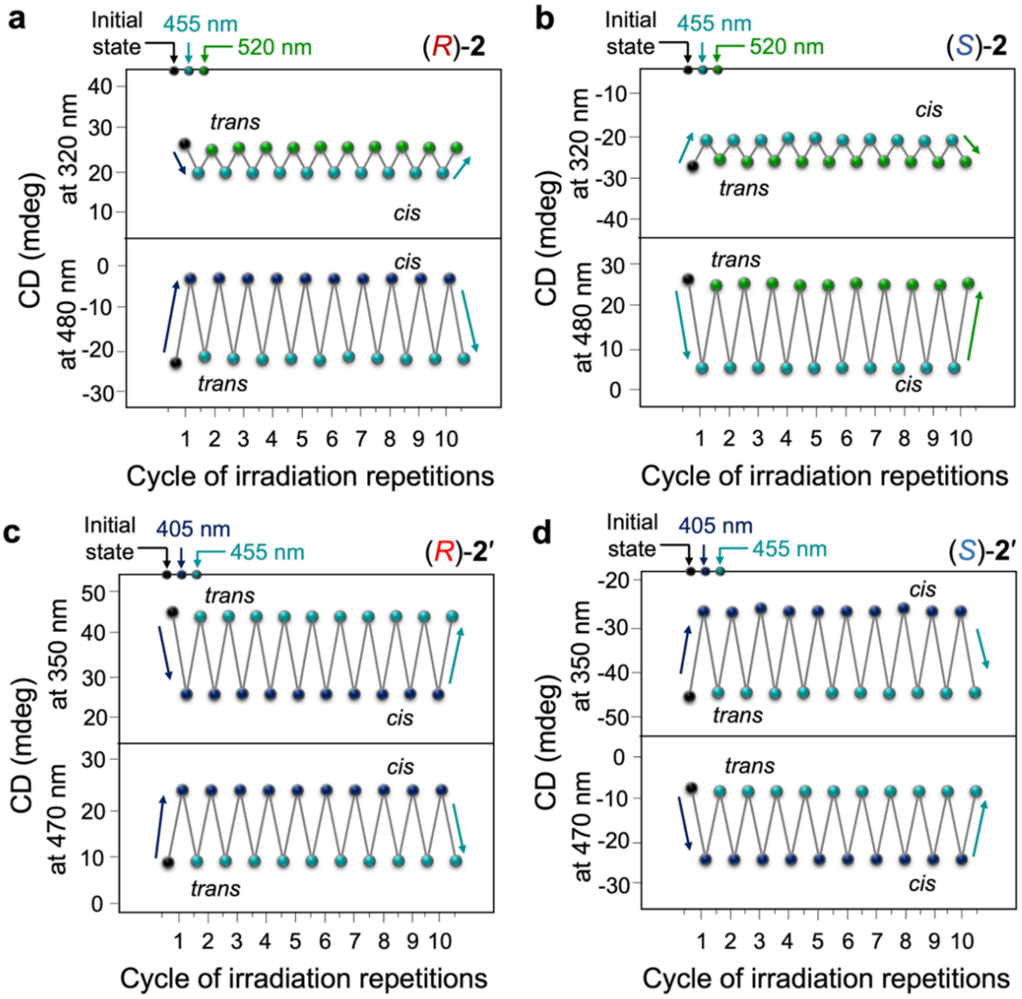

Figure S16. Photoswitchable behavior of the CD signals of chiral photoswitches (a) (*R*)-**2**, (b) (*S*)-**2**, (c) (*R*)-**2′** and (d) (*S*)-**2′**, all at 0.5 mM in 1,4-dioxane, (monitoring wavelengths: 350 and 470 nm for **2**, and 320 and 460 nm for **2′**) after repetitions of 10 irradiation cycles. (light illumination: 12 min)

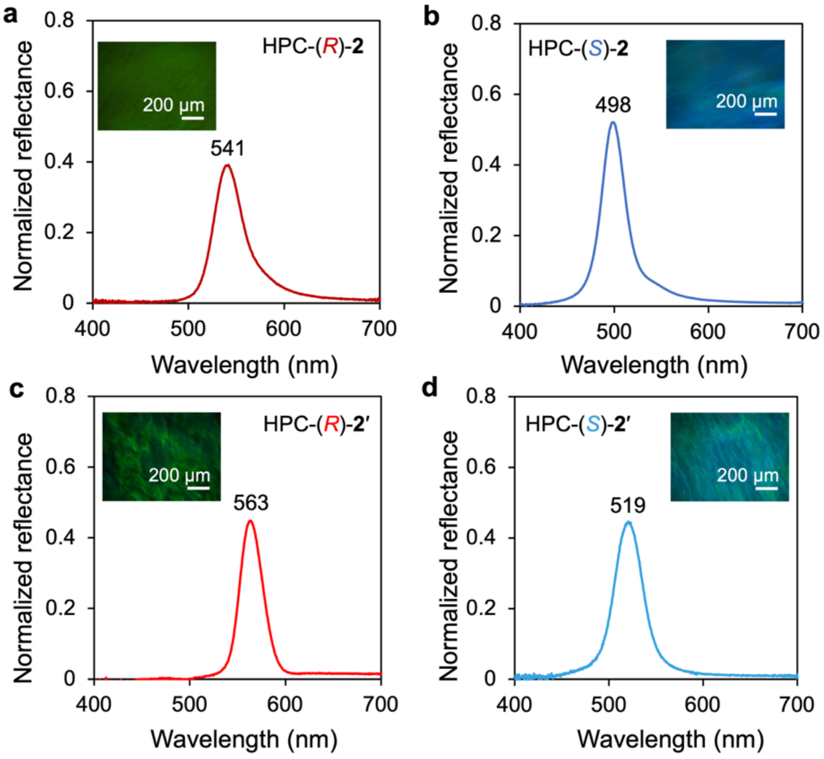

Figure S17. Reflection spectra with insets displaying OM images of HPC mesophases (72 wt.% in DMSO) containing 0.1 wt.% of photoswitch (*R*)-**2** (a), (*S*)-**2** (b), (*R*)-**2′** (c), and (*S*)-**2′** (d).

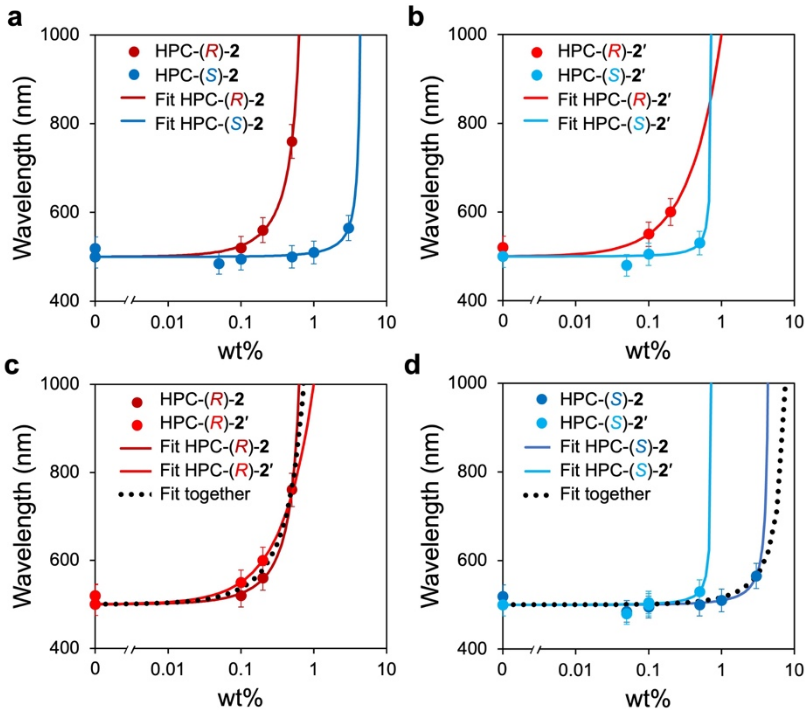

Figure S18. Variation of the reflection peak wavelength of HPC mesophase (72 wt.% in DMSO) as a function of the concentration of added chiral photoswitches (a) (*R*)-**2** and (*S*)-**2** and (b) (*R*)-**2′** and (*S*)-**2′**, (c) (*R*)-**2** and (*R*)-**2′** and (d) (*S*)-**2** and (*S*)-**2′**. Lines correspond to fitting $\lambda(c) = \lambda(0)(1 + \kappa\, c)/(1 + \chi\, c)$, where c is the photoswitch concentration (see discussion below). The black dotted line corresponds to the fit assuming **2** and **2′** have the same behavior in order to account for the insufficient dataset.

The reflection peak wavelength and the pitch are related by $\lambda = n\, p$, where $n \approx 1.486$ is the average refractive index of the mesophase. At such low dopant concentration, the effect of dopant concentration on the mesophase refractive index is negligible, so the variation of the peak wavelength with dopant concentration results from the pitch dependence, which is expected to scale as:

$$p = 2\pi\, K_{22}(c)\,/K_t(c) \quad \text{(S2)}$$

where $K_{22}(c)$ is the twist elastic constant of the mesophase and $K_t(c)$ its chiral strength, which are both dependent on the concentration c of added dopant.

Assuming that these constants scale linearly with $c$for small dopant concentrations, it follows that

$$K_{22}(c) = K_{22}{}^{HPC}\,(1 + \kappa\, c) \quad \text{(S3)}$$

$$K_t(c) = K_t{}^{HPC}\,(1 + \chi\, c) \quad \text{(S4)}$$

where $K_{22}{}^{HPC}$ and $K_t{}^{HPC}$ correspond to the pure HPC mesophase in absence of added dopant. Combining the equations (S2-S4), the reflected peak wavelength dependence with c can be expressed as follows:

$$\lambda(c) = \lambda(0)(1 + \kappa\, c)/(1 + \chi\, c) \quad \text{(S5)}$$

This formula was used for the fitting shown in **Figure S18,** with the fitting parameters in **Table S6**.

Table S6. Fitting parameters ($\boldsymbol{\kappa}$, $\boldsymbol{\chi}$) for the *R* and *S* enantiomers of **2** and **2′** used in the Figure S18, expressed in such units that c is in wt.% (i.e. c = 1 for 1 wt.%). The lines $\bar{\boldsymbol{x}} = (\boldsymbol{x_R} + \boldsymbol{x_S})/\boldsymbol{2}$, $\Delta \boldsymbol{x} = (\boldsymbol{x_R} - \boldsymbol{x_S})/\boldsymbol{2}$ and $\Delta \boldsymbol{x}/\bar{\boldsymbol{x}} = (\boldsymbol{x_R} - \boldsymbol{x_S})/(\boldsymbol{x_R} + \boldsymbol{x_S})$ correspond to linear symmetric and antisymmetric combinations of these parameters, as well as their relative ratio. Values for **2** and **2′** only are subject to high uncertainty (in grey font) and are only reported for completeness, while more robust values are estimated from merging the two datasets (**2**, **2′**) into a single one (labelled **2** + **2′**) to increase the reliability of the fit, and correspond to the two dotted lines (for *R* and *S*) in Figure S18.

Molecule	Enantiomer	$x = \kappa$	$x = \chi$
**2**	x_R	-0.71	-1.15
	x_S	-0.20	-0.22
	$\bar{x}$	-0.45	-0.68
	Δx	-0.25	-0.47
	$\Delta x/\bar{x}$	0.56	0.68
**2′**	x_R	1.00	0.00
	x_S	-1.28	-1.32
	$\bar{x}$	-0.14	-0.66
	Δx	1.14	0.66
	$\Delta x/\bar{x}$	-8.10	-1.00
**2 + 2′**	x_R	-0.01	-0.69
	x_S	-0.07	-0.10
	$\bar{x}$	-0.04	-0.40*
	Δx	0.03	-0.29**
	$\Delta x/\bar{x}$	-0.80	0.74

*The strong achiral coupling between HPC and (**2**, **2’**) is highlighted by $\bar{\boldsymbol{\chi}} = \frac{(\chi_R+\chi_S)}{2} = \boldsymbol{-0.40}$;

The strong chiral coupling between HPC and (2**, **2’**) is highlighted by $\boldsymbol{\Delta\chi} = \frac{(\chi_R-\chi_S)}{2} = \boldsymbol{-0.29}$.

11. Photoisomerization effect on the polarization state of the HPC-1 reflection

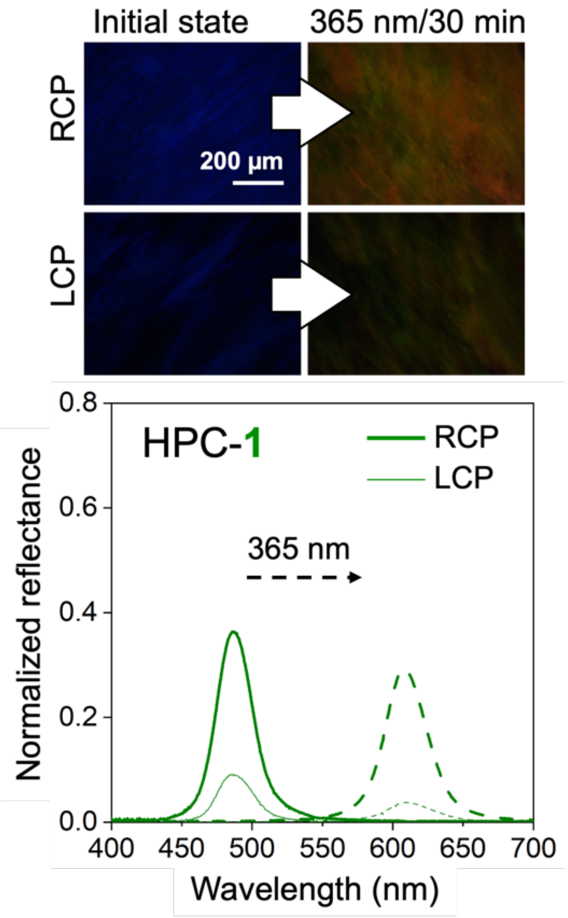

Figure S19. POM images and the corresponding change in reflection spectra of HPC-**1** mesophase (72 wt.% in DMSO) containing 0.1 wt.% of the photoswitch **1** upon 365 nm light illumination. Solid lines for the initial states. Dashed lines for the PSSs after light illumination at 365 nm.

12. Photoisomerization effect on the polarization state of the HPC reflection with 2 and 2′

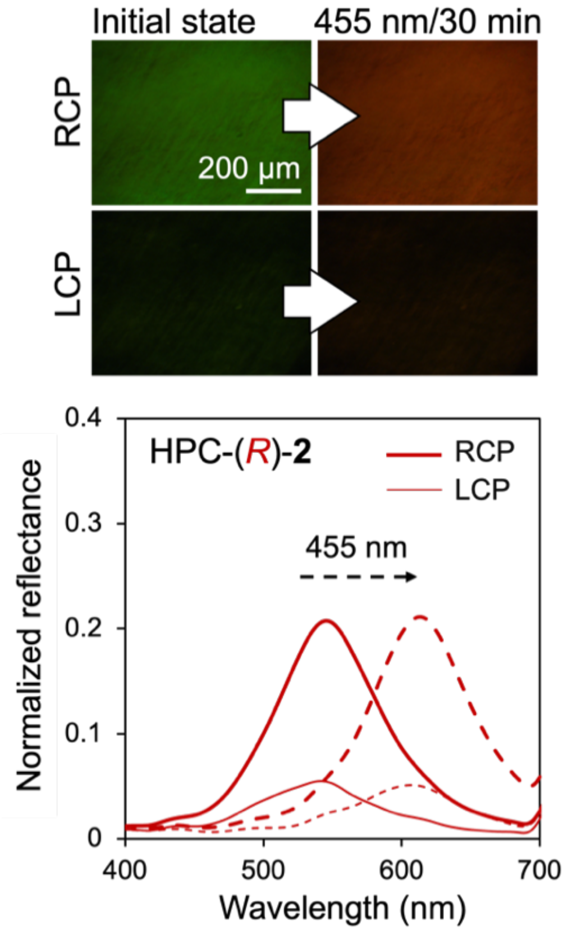

Figure S20. POM images and the corresponding change in reflection spectra upon 455 nm light illumination of an HPC mesophase (72 wt.% in DMSO) containing 0.1 wt.% of photoswitch (*R*)-**2**. Solid lines for the initial states. Dashed lines for the PSSs after light illumination at 455 nm.

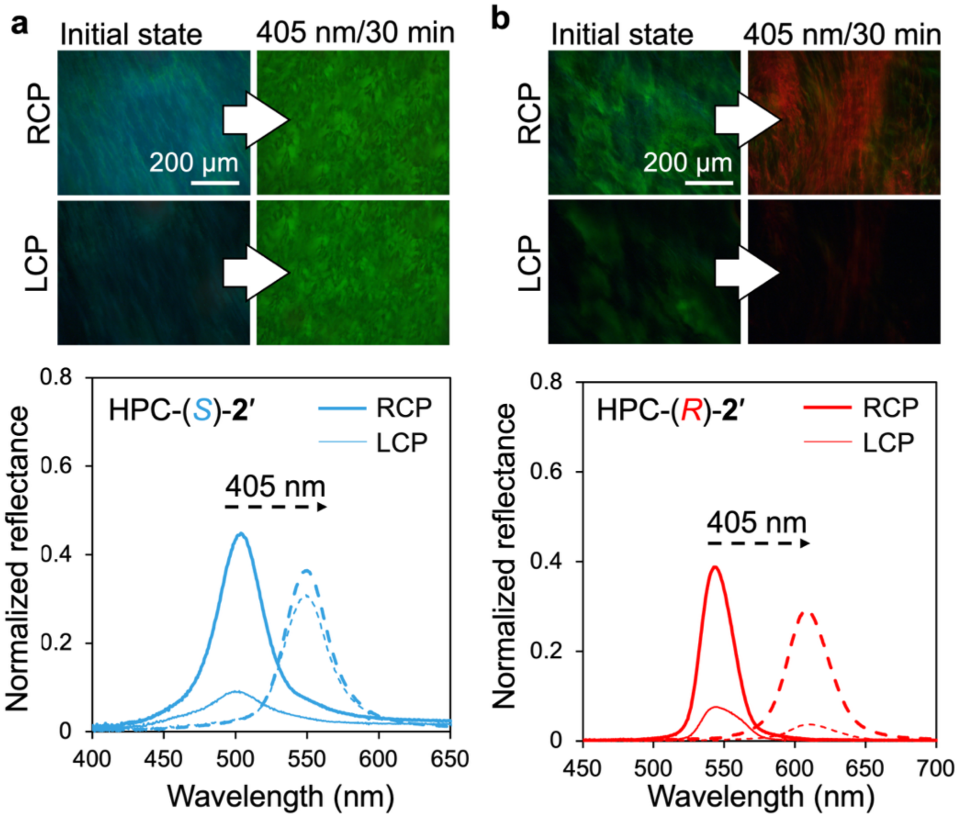

Figure S21. POM images and the corresponding change in reflection spectra upon 405 nm light illumination of HPC mesophase (72 wt.% in DMSO) containing 0.1 wt.% of the photoswitch (*S*)-**2′** (a) and (*R*)-**2′** (b). Solid lines for the initial states. Dashed lines for the PSSs after light illumination at 405 nm.

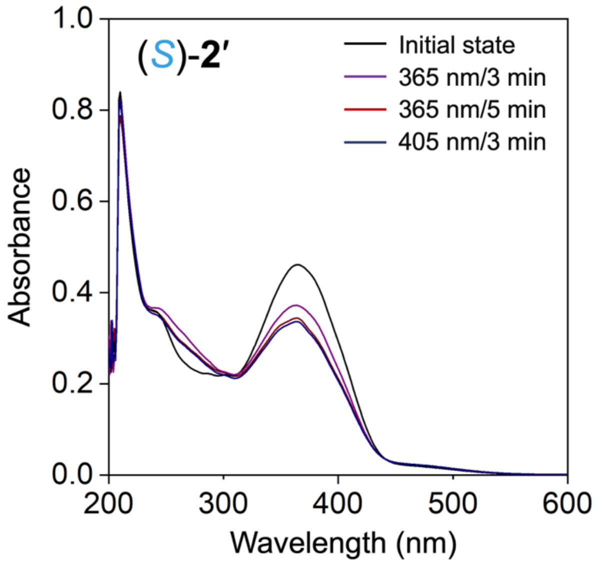

Figure S22. UV-Vis absorption spectra of the photoswitch (S)-**2′** in 1,4-dioxane (5 μM) after 365 nm compared to 405 nm illumination.

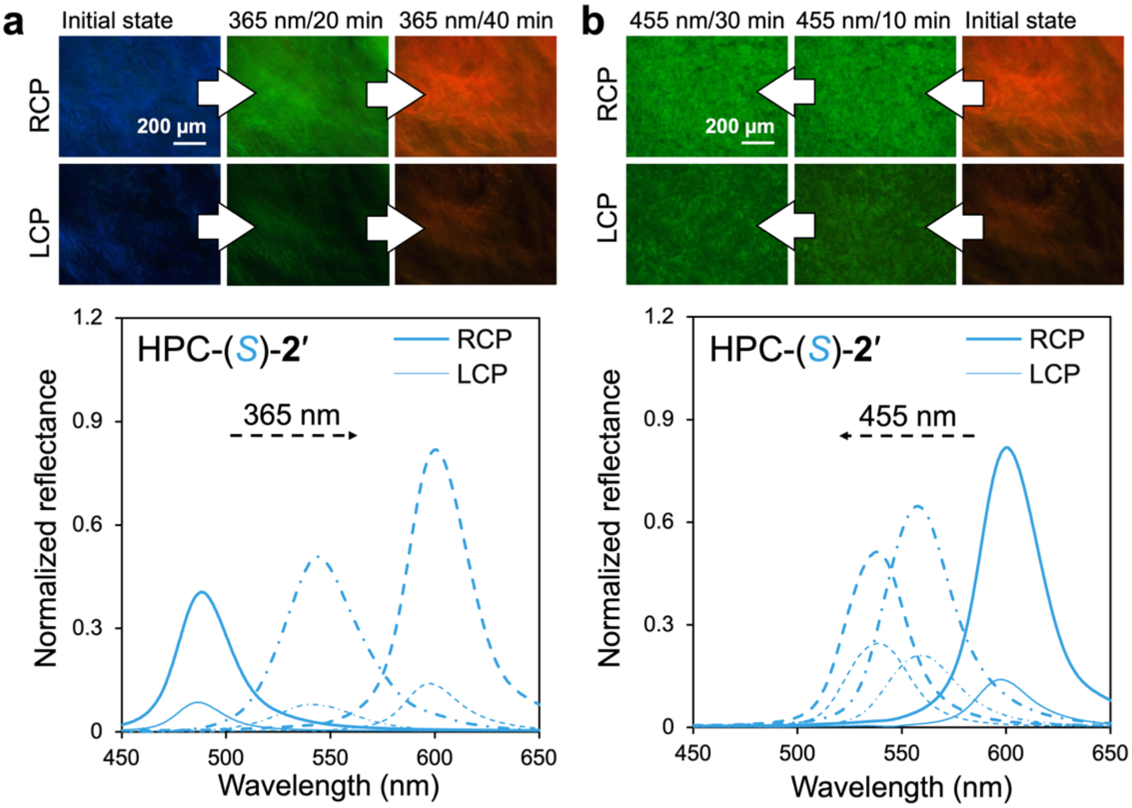

Figure S23. HPC mesophase (72 wt.% in DMSO) containing 0.1 wt.% of the photoswitch (*S*)-**2′**. (a) POM images and the corresponding reflection spectra for initial states (solid lines) and upon 365 nm light illumination (after 20 min: dash-dotted line; after 40 min: dashed lines). (b) POM images and the corresponding reflection spectra for PSSs (solid lines) and the back isomerization after 455 nm illumination (after 10 min: dash-dotted line; after 30 min: dashed lines).

13. Thermo-responsive behavior for a full color change from blue to red

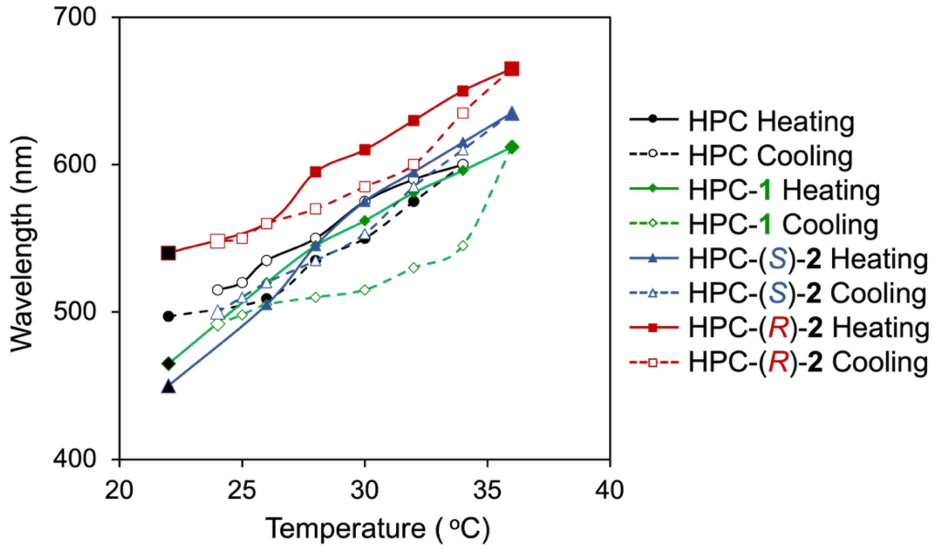

Figure S24. The change in maximum reflection wavelength for HPC, HPC-**1**, HPC-(*S*)-**2**, HPC-(*R*)-**2** in heating (solid lines) and cooling (dashed lines) cycles. HPC mesophase (72 wt.% in DMSO) with photoswitch (0.1 wt.%).

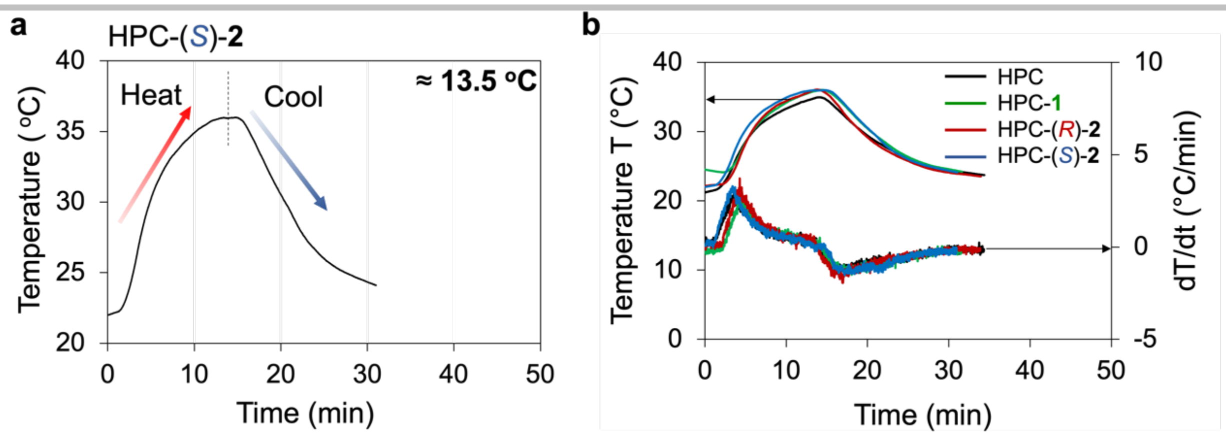

Figure S25. a) Monitored temperature inside an HPC-(*S*)-**2** film upon a heating-cooling cycle (leading to color change from blue to red in Figure S26). b) Comparison of monitored temperature inside HPC films between different photoswitches upon heating-cooling cycles, showing comparable heating and cooling rates for all of them (with a maximum heating rate of dT/dt ≈ +3 °C/min and a maximum cooling rate of dT/dt ≈ -1.5 °C/min), ruling out this parameter as the primary cause of their different behaviors.

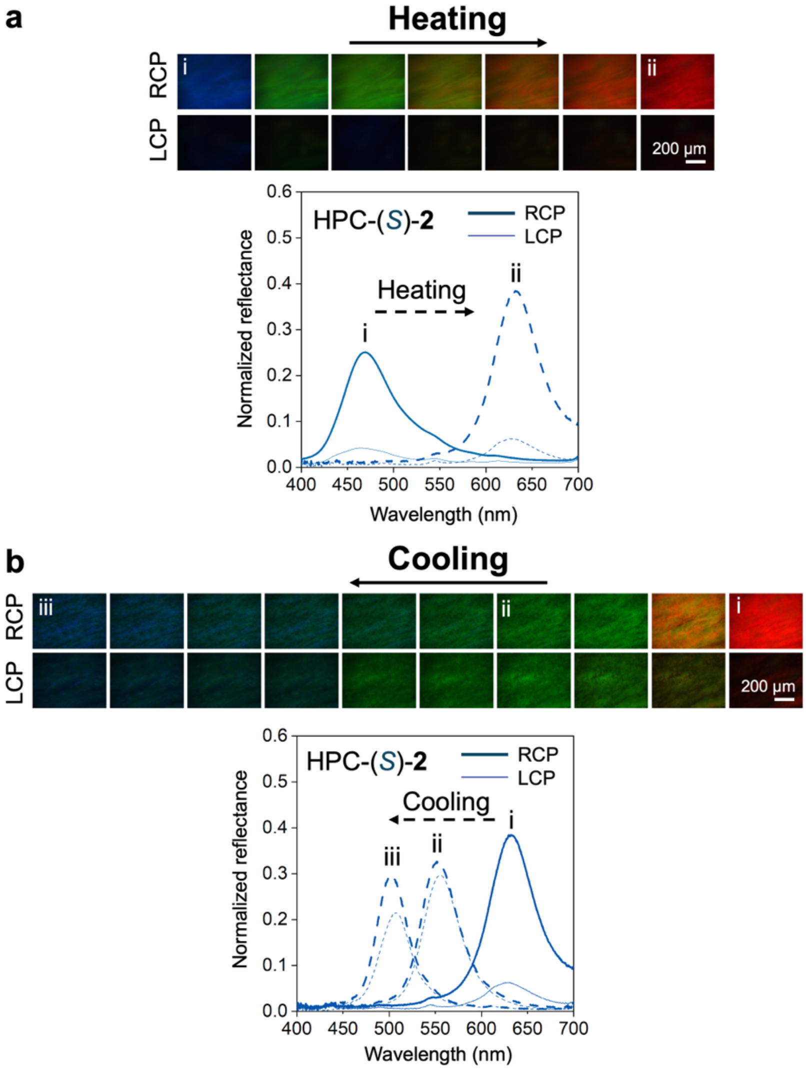

Figure S26. POM images and the corresponding change in reflection spectra of HPC mesophase (72 wt.% in DMSO) containing 0.1 wt.% of the photoswitch (*S*)-**2** upon heating (a) and for the cooling cycle (b). Straight lines for the initial states. Dashed lines upon heating or cooling.

14. Comparative optical analysis of the thermo- and photo-responsive behaviors

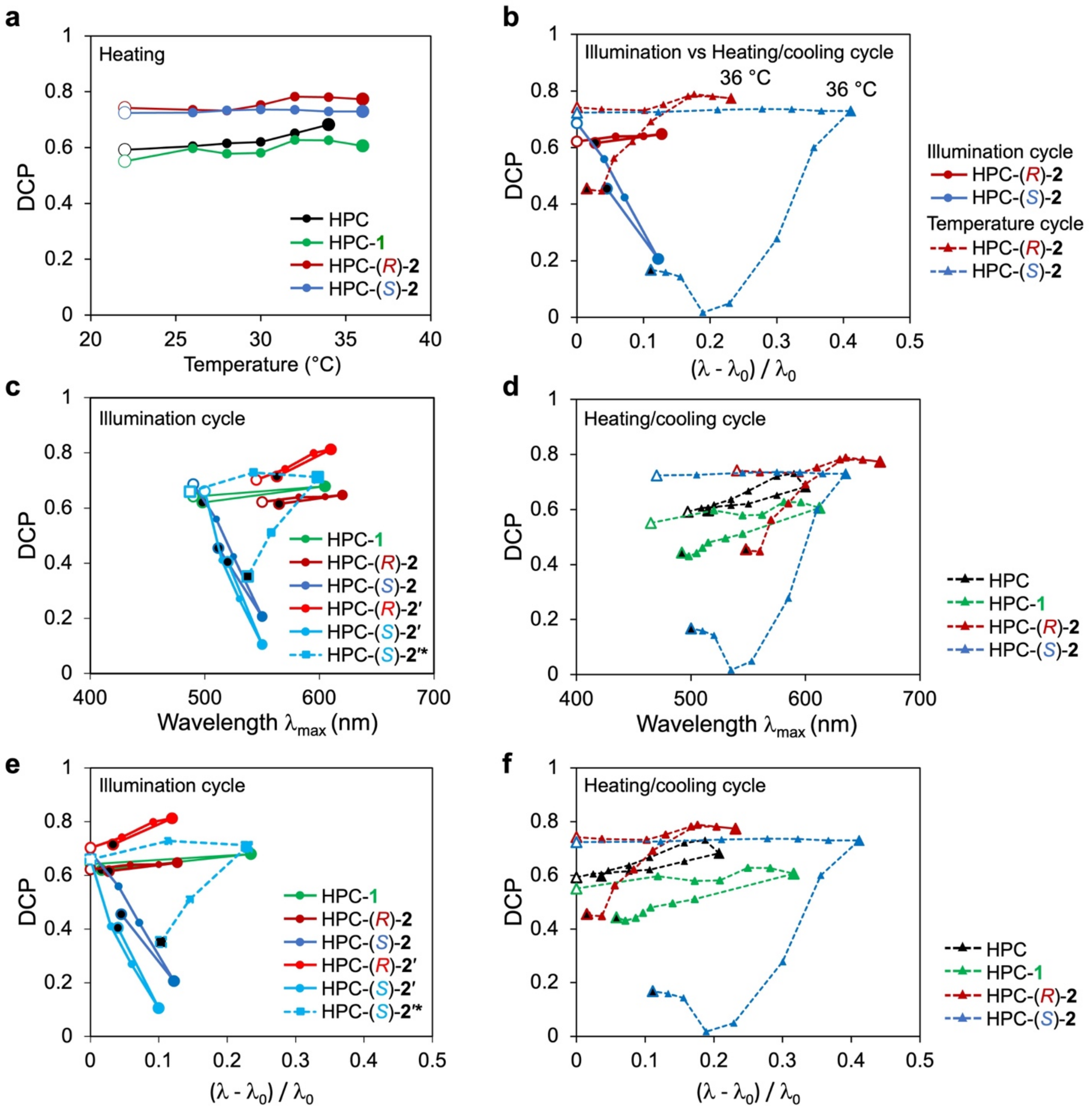

Figure S27. a) Degree of circular polarization (DCP) for HPC, HPC-**1**, HPC-(*R*)-**2**, and HPC-(*S*)-**2** and **2′** upon heating for $\Delta T \approx 13.5\,^{\circ}C$. See **Figure 3d** for DCP change with temperature upon cooling. b) Comparison of DCP vs relative redshift for HPC-(*R*)-**2**, and HPC-(*S*)-**2** between illumination (circles) and heating-cooling (triangles) cycles. Initial points are displayed in open symbols, point with highest redshift are in enlarged full symbols, final points are filled in black. c-f) Comparison of DCP vs absolute (c,d) and relative redshift (e,f) during illumination cycle (c,e) or heating-cooling cycle (d,f) for HPC with different photoswitching molecules. The illumination wavelength used for the *trans-cis* transition was 455 nm for **2**, both 405 nm and 365 nm for **2′**. The **2′** sample illuminated at 365 nm is denoted here as **2′***. The reverse *cis-trans* transition was induced using 520 nm for **2** and 455 nm for both **2′** and **2′***.

Supporting discussion

The dramatically different results for (*S*)- and (*R*) enantiomers of **2** and **2′** can be attributed to the change in interaction between HPC and the molecular switches. Notably, while (*R*)-**2** and (*R*)-**2′** in their *trans* configuration (before isomerization) have a larger equilibrium pitch than their (*S*) enantiomeric counterparts (Figure S27c), *trans-cis* isomerization of these switches leads to a similar relative red-shift $\Delta\lambda_{\max}/\lambda_{\max}$ (Figure S27e), suggesting that it is caused either by a decrease of the symmetric chiral strength term $\bar{\chi}$ or by an increase of the symmetric twist elastic term $\bar{\kappa}$ (as defined in Section S10), both of which could be related to the change of the geometry of the molecule.

To grasp the ability for complex geometric shapes to densely pack and influence each other's alignment, the similarity of the isoperimetric quotient (IPQ) of their shape (defined in 3D as $36\pi V^2/S^3$, where V and S stands for the shape volume and surface) has been proposed as a relevant metric for chirality transfer between the dopant molecules and the main mesogen molecules (here the HPC): the action of the dopant onto the mesogen should be optimum when they share a similar IPQ.[S9],[S10] In this study, the IPQ was evaluated based on their convex hull shape (defined as $36\pi\ V_{ch}^2\ /\ S_{ch}^3$), with the idea that it can arguably be a more relevant metric to better account for how molecular chiral dopants can fit within less flexible and densely aligned HPC chains (assuming long HPC molecules have a low IPQ << 1).
For the case of HPC-**1**, we observe that this convex hull IPQ increases upon isomerization (*trans*-**1**: 0.434, *cis*-**1**: 0.619), mainly due to an apparent convex hull volume increase of *ca* +20%. Since the bare volume of the molecule does not really change, it is mostly due to the volume increase arising from voids within the molecule, reducing its overall compacity (-16.5%, see Table S2). From Figure S5, we can see that adding 0.1% of *trans*-**1** to HPC causes a negligible pitch increase (with a reflection peak more or less unaffected around 495±5 nm), but upon its isomerization to *cis*-**1** it causes an important redshift (490 nm → 608 nm, Figure S19), comparable to the redshift obtained upon adding as much as 2% of *trans*-**1** to HPC (605 nm, see Figure S5). The cross-comparison between these values and the molecular parameters of Table S2 suggests that the redshift upon *trans-cis* isomerization is driven by a compacity decrease, in spite of an increase of convex hull IPQ.

For the case of HPC-**2** and HPC-**2'**, and given that the convex hull isoperimetric quotients of the (*S*)-**2** and (*S*)-**2′** (and their respective enantiomers) do not meaningfully change upon trans-cis isomerization (*trans*-(*S*)-**2**: 0.629, *cis*-(*S*)-**2**: 0.636; *trans*-(*S*)-**2′**: 0.467, *cis*-(*S*)-**2′**: 0.423, see Table S4 and Table S5), it is likely that its overall anisometric shape and thus its ability to intercalate within the aligned HPC polymeric chains remains comparable after UV illumination. The inspection of the compacity change upon *trans-cis* isomerization indicates a net increase (with (*S*)-**2**: +6.7% and (*S*)-**2'**: +17.7%, see Table S4-S5), and their largest interatomic distances (within the molecule) decreases, suggesting both molecules **2** and **2'** get more bulky. Finally, the dihedral angles CNNC switch from achiral for *trans*- (180° for both **2** and **2'**) to a chiral value for *cis*- (13° for both **2** and **2'**), while the CCCC dihedral angle switches from near achiral (89° for **2**, 92° for **2'**) to much clearly chiral values (74° for **2**, 73° for **2'**). Under such conditions, we can expect that (*S*)-**2** and (*S*)-**2′**, as well as their enantiomeric counterparts, mainly interact through a more negative twisting strength K_t upon *trans-cis*-isomerization. While this term is already significantly negative for the (*R*) enantiomers, it is much weaker for the (*S*) enantiomers, and this could possibly explain why the *trans-cis* isomerization induced upon visible light illumination causes a much

more abrupt jump in χ_L, leading not only to a significant redshift but also a disruption of the helicoidal order, a distortion of the local domains and a drop in DCP. It is unclear, however, whether the distribution of these photoswitches throughout the mesophase remains homogeneous or whether it locally clusters, resulting in inhomogeneous local twisting strengths.

15. Photomasking patterns and writing with UV light beam applications for HPC-3

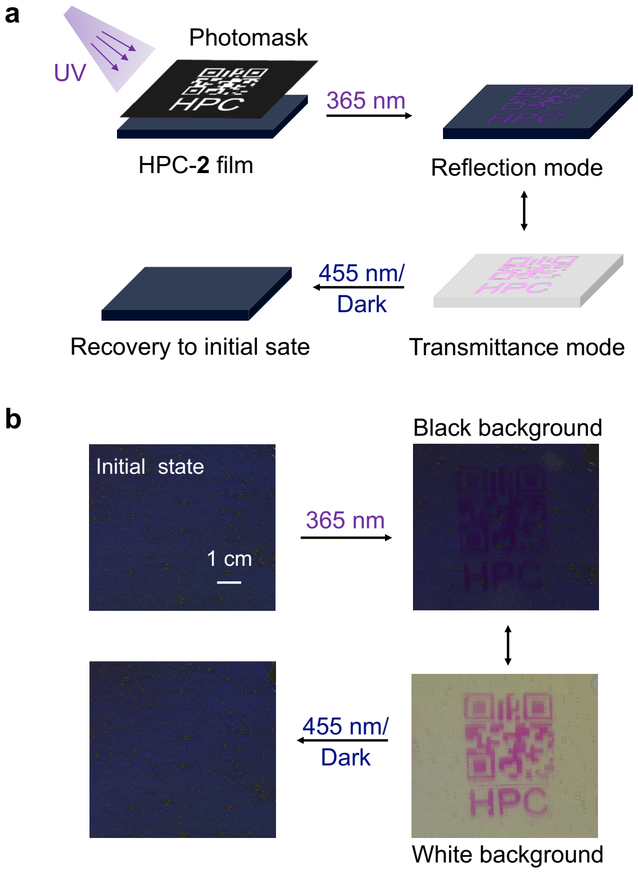

Figure S28. (a) Schematic illustration for QR code patterns generated upon UV light illumination by photomask for 10 min. (b) Photographs of HPC mesophase (72 wt.% in DMSO) containing 0.1 wt.% of photoswitch **3** before and after light irradiation, imaged on black or white backgrounds. Recovery to the initial state was achieved with 455 nm visible light illumination for 10 min or by keeping it in the dark for 20 minutes.

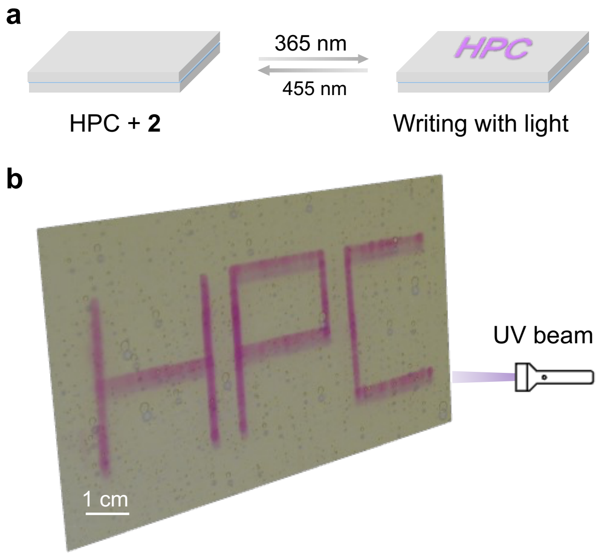

Figure S29. (a) Writing with UV light beam on HPC-**3**. (b) Light-sensitive photonic display of HPC-**3** (72 wt.% in DMSO) containing 0.1 wt.% of photoswitch **3** after cumulative exposure to 365 nm light for 20 minutes during repeated writing.

16. ^{1}H- and ^{13}C-NMR spectra

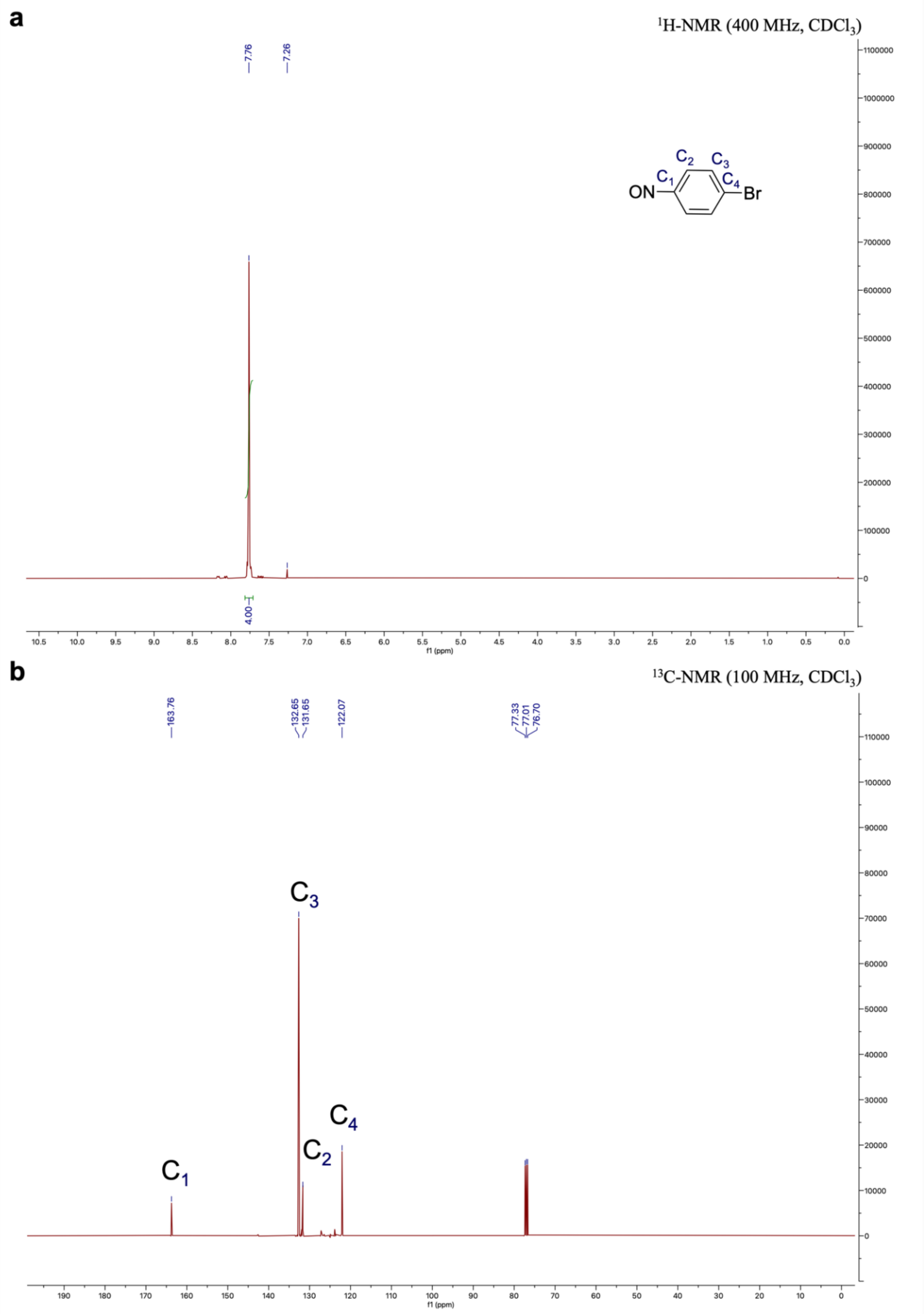

Figure S30. ^{1}H-NMR (a) and ^{13}C-NMR (b) spectra of **NOPhBr** in $CDCl_3$.

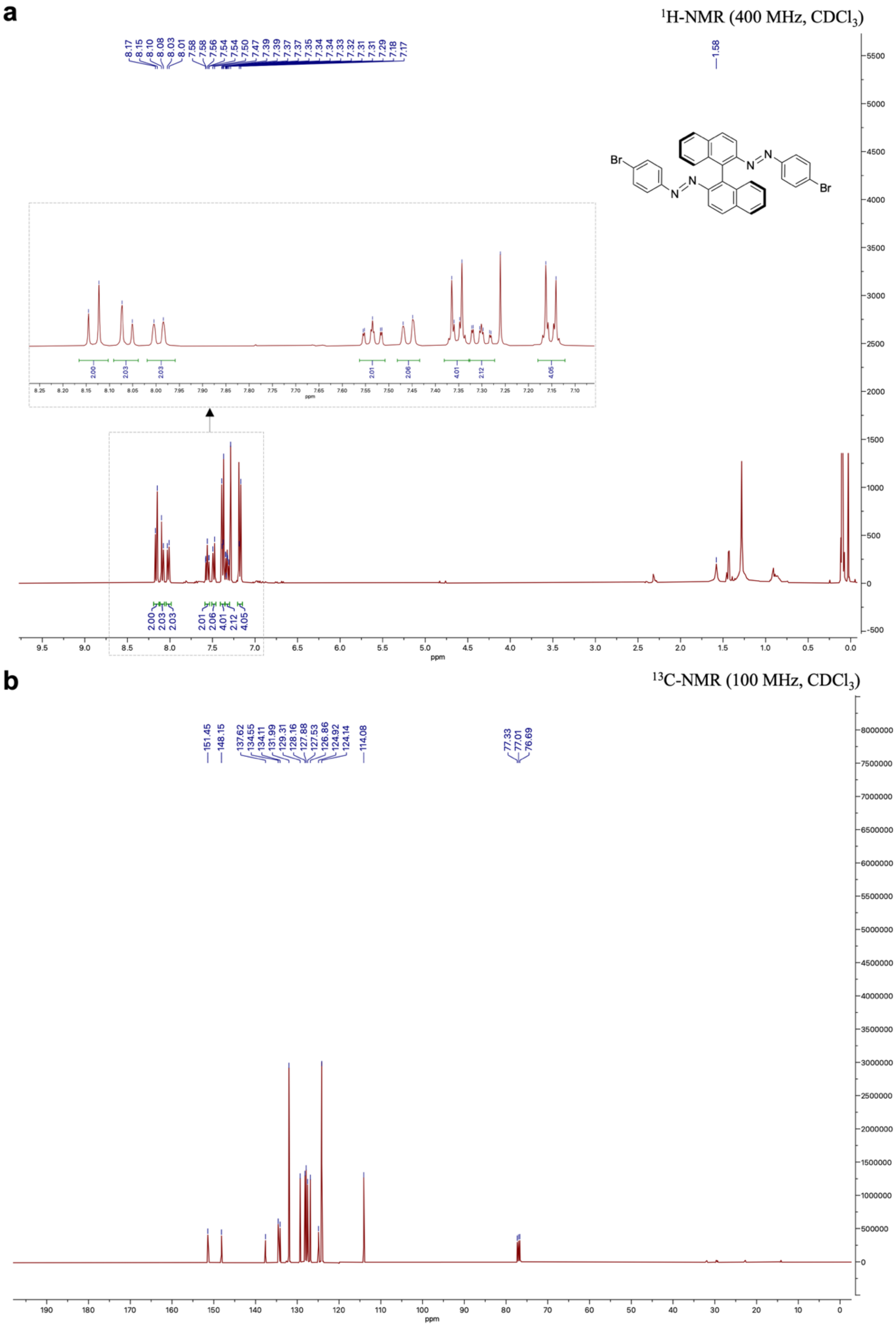

Figure S31. ^{1}H-NMR (a) and ^{13}C-NMR (b) spectra of (*R*)-**DiBrAzo** in $CDCl_3$.

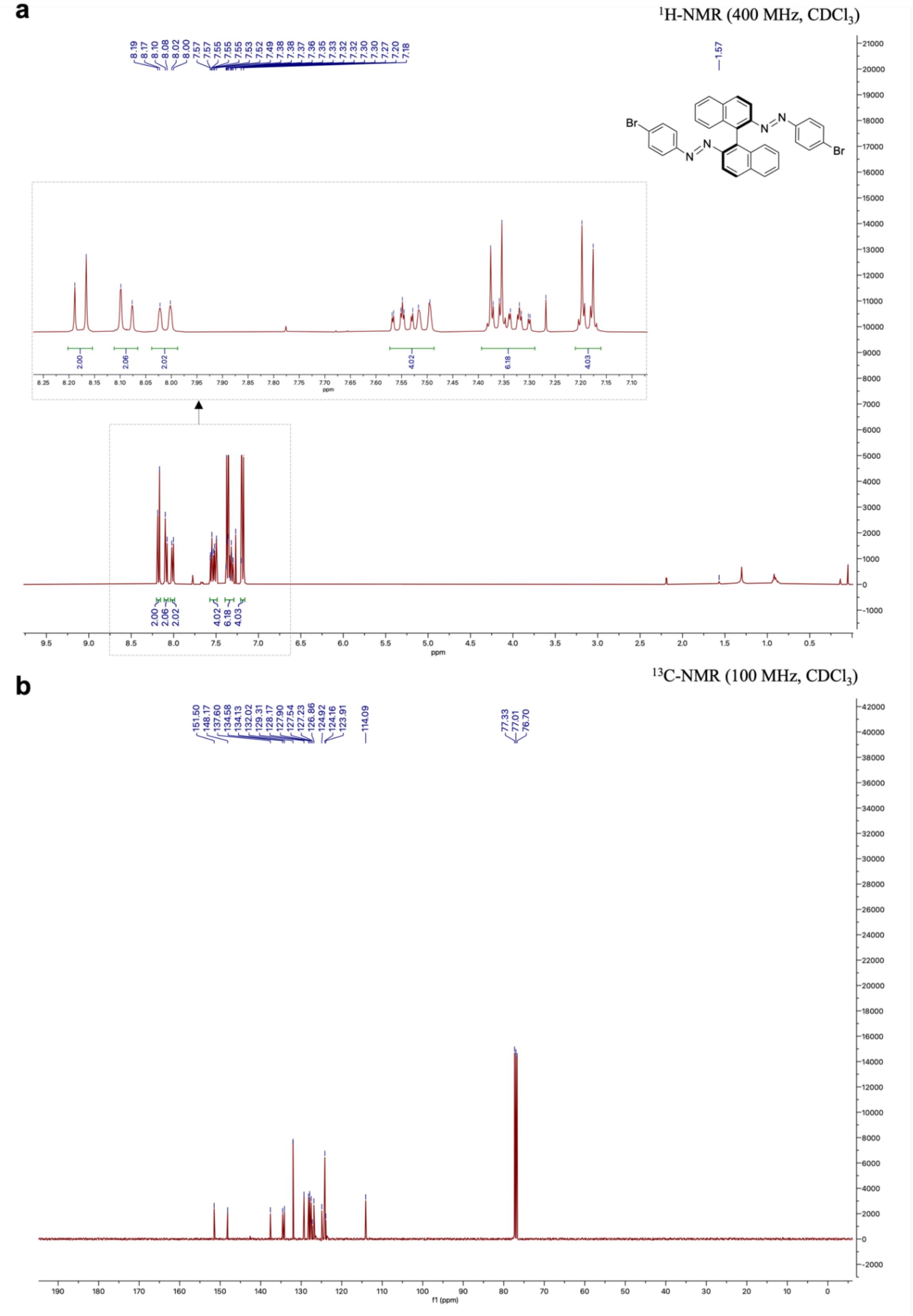

Figure S32. ^{1}H-NMR (a) and ^{13}C-NMR (b) spectra of (*S*)-**DiBrAzo** in CDCl$_3$.

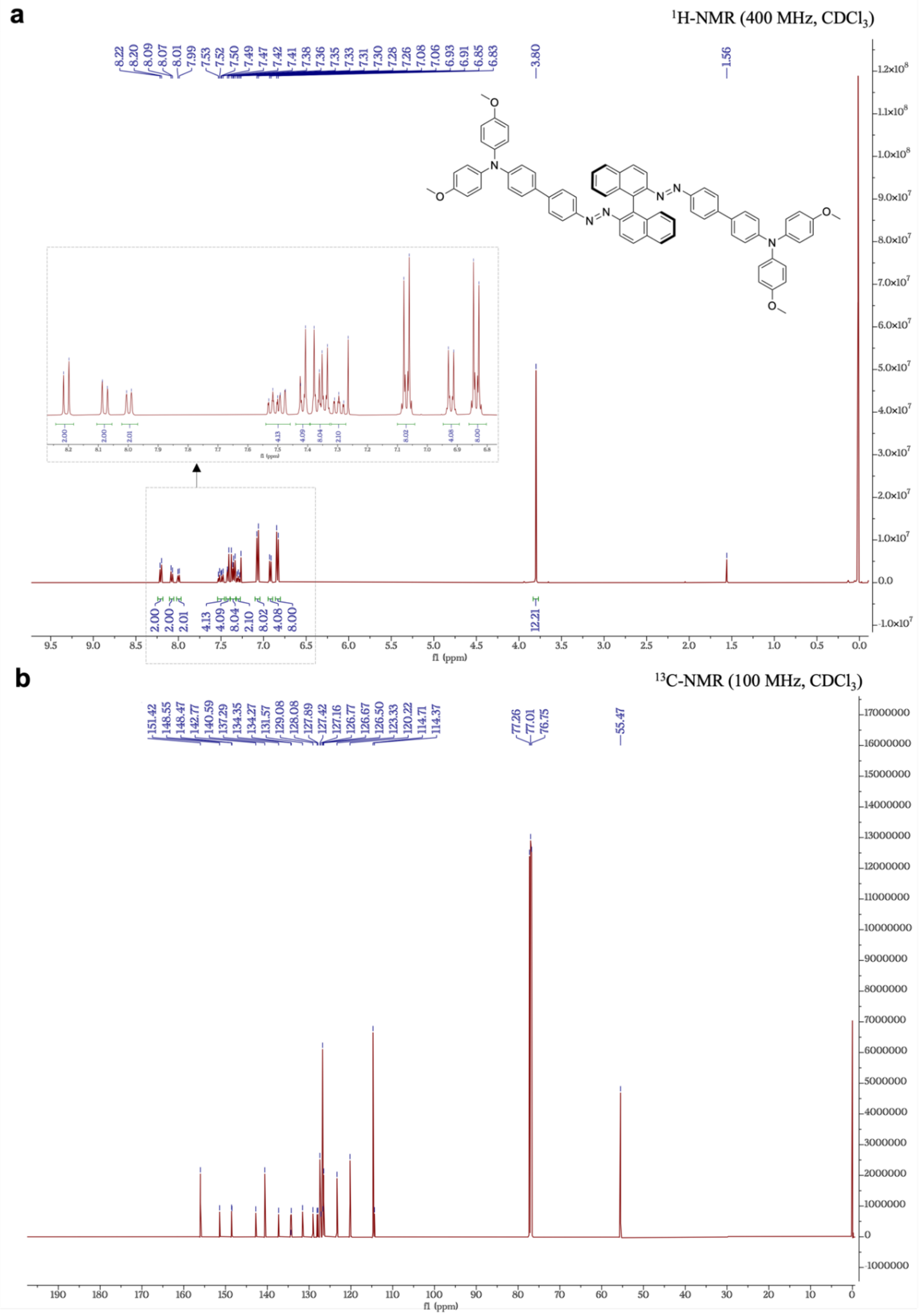

Figure S33. ^{1}H-NMR (a) and ^{13}C-NMR (b) spectra of (*R*)-**2** in CDCl$_3$.

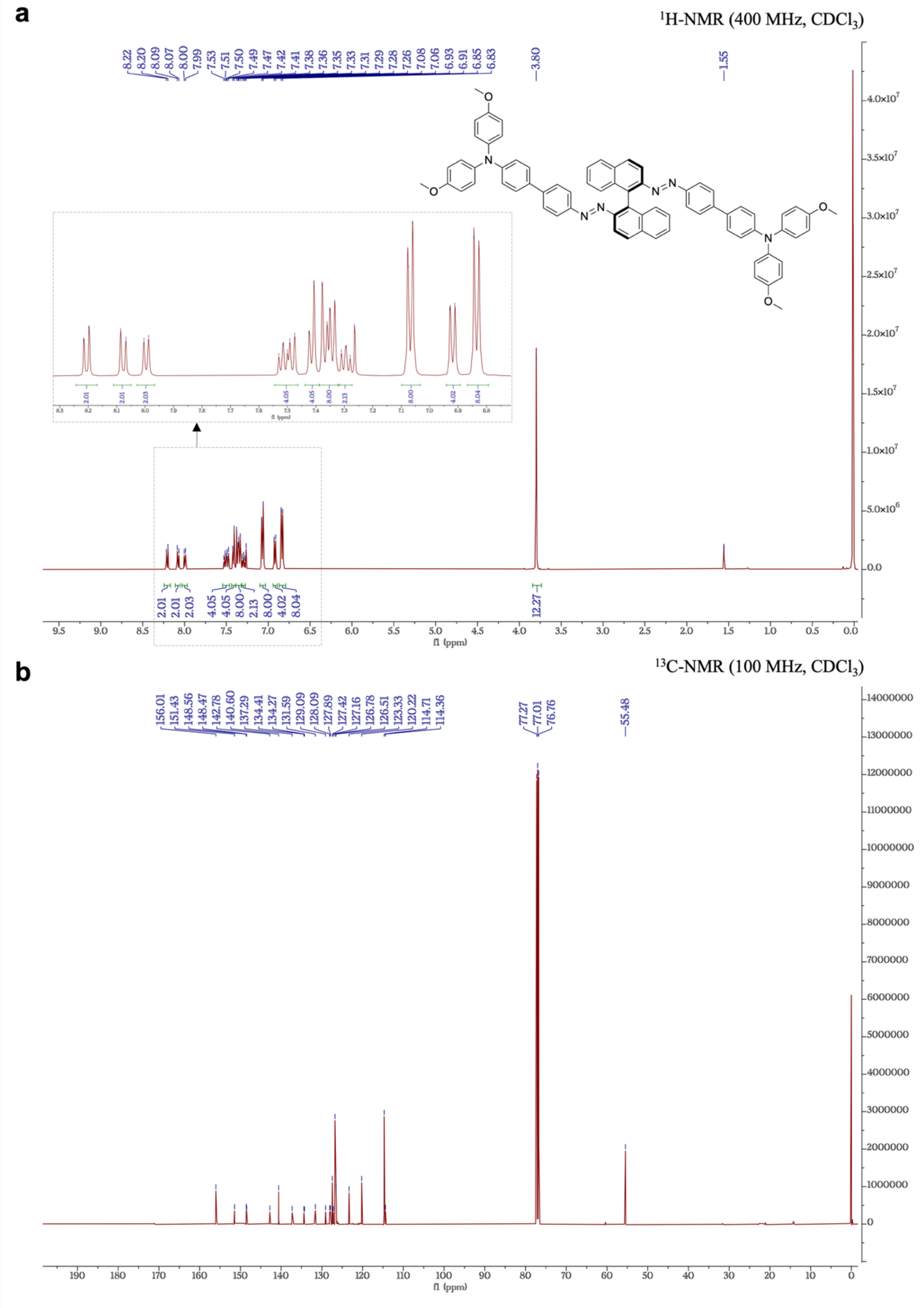

Figure S34. ^{1}H-NMR (a) and ^{13}C-NMR (b) spectra of (*S*)-**2** in CDCl$_3$.

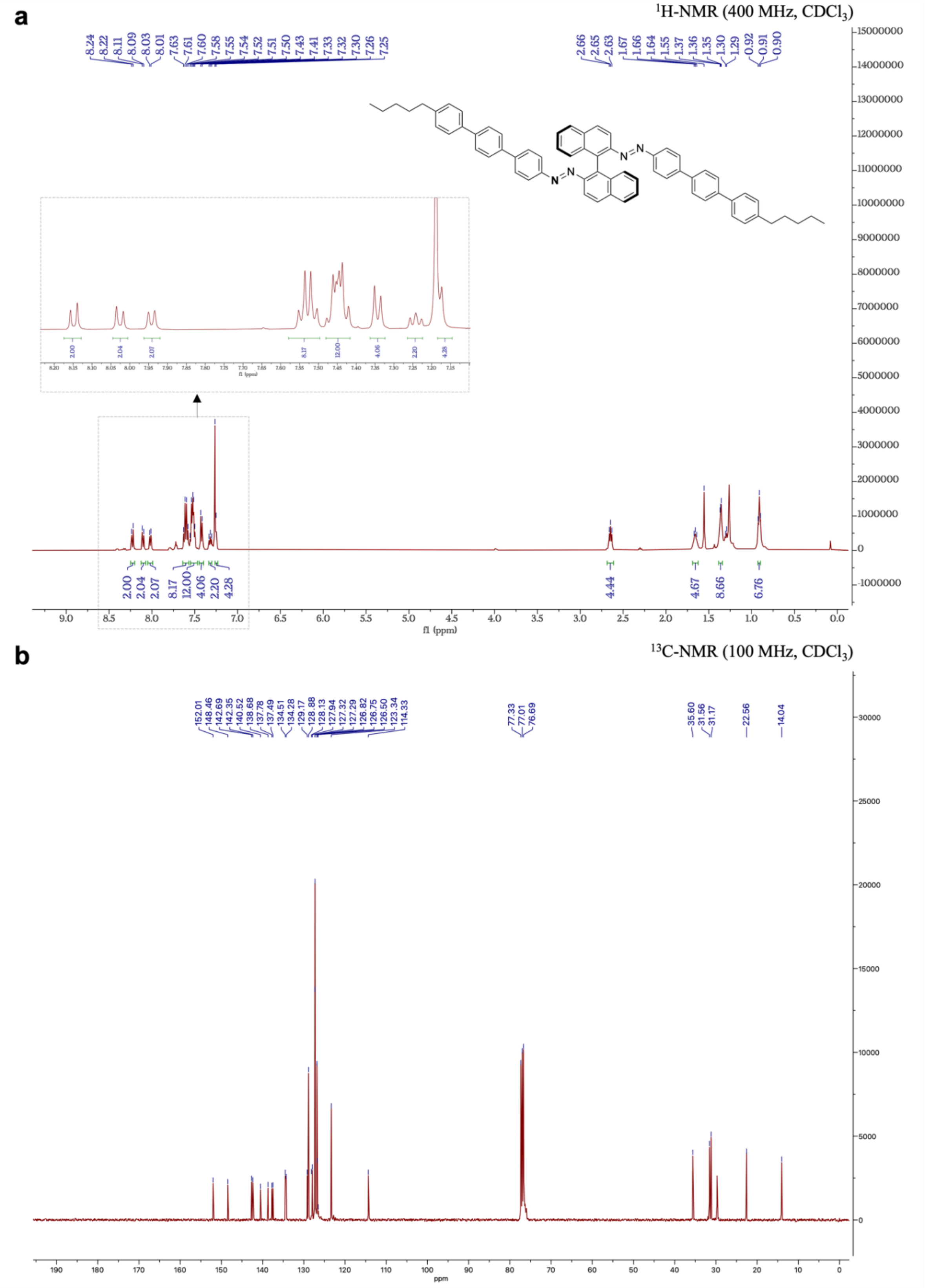

Figure S35. ^{1}H-NMR (a) and ^{13}C-NMR (b) spectra of (*R*)-**2′** in $CDCl_3$.

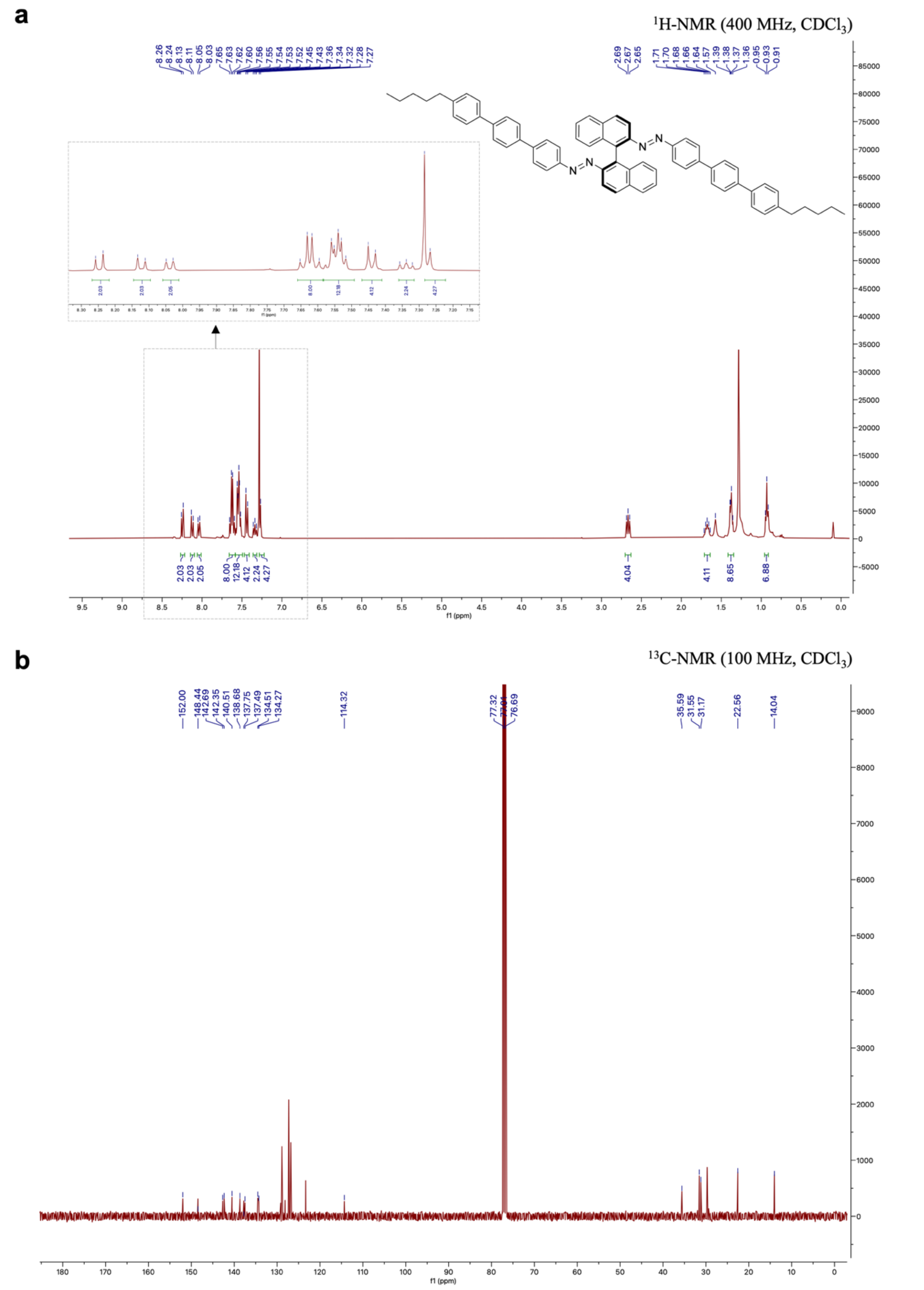

Figure S36. ^{1}H-NMR (a) and ^{13}C-NMR (b) spectra of (*S*)-**2′** in $CDCl_3$.

17. Supplementary references

[S1] X. Zhang, F. Hassan, H. K. Bisoyi, H. Wang, Z. Zhou, and Q. Li, *Mater. Chem. Front.* **2022,** *6*, 3698-3705.

[S2] H. Ren, I. O. Sodipo, A. G. Dumanli, *ACS Appl. Polym. Mater.* **2025**, *7*, 4093–4098.

[S3] C. L. C. Chan, I. M. Lei, G. T. van de Kerkhof, R. M. Parker, K. D. Richards, R. C. Evans, Y. Y. S. Huang, S. Vignolini, *Adv. Funct. Mater.* **2022**, *32*, 2108566.

[S4] S. R. Seyednejad, M. R. Mozaffari, *Phys. Rev. E* **2021**, *104*, 014701.

[S5] X. Li, T. Yanagimachi, C. Bishop, C. Smith, M. Dolejsi, H. Xie, K. Kurihara, P. F. Nealey, *Soft Matter* **2018**, *14*, 7569–7577.

[S6] V. Nazarenko, O. Boiko, H.-S. Park, O. Brodyn, M. Omelchenko, L. Tortora, Y. A. Nastishin, O. Lavrentovich, *Phys. Rev. Lett.* **2010**, *105*, 017801.

[S7] H. Lange, F. Schmid, *Eur. Phys. J. E* **2002**, *7*, 175–182.

[S8] Y. Onogi and Y. Nishijima, *Kobunshi Ronbunshu* **1986**, *43*, 223-229.

[S9] A. Nemati, L. Querciagrossa, C. Callison, S. Shadpour, D. P. Nunes Gonçalves, T. Mori, X. Cui, R. Ai, J. Wang, C. Zannoni, *Sci. Adv.* **2022**, *8*, eabl4385.

[S10] T. G. Parton, R. M. Parker, G. T. Van De Kerkhof, A. Narkevicius, J. S. Haataja, B. Frka-Petesic, S. Vignolini, *Nat. Commun.* **2022**, *13*, 2657.
